# Recent advances in the valorization of plant biomass

**DOI:** 10.1186/s13068-021-01949-3

**Published:** 2021-04-23

**Authors:** Peng Ning, Guofeng Yang, Lihong Hu, Jingxin Sun, Lina Shi, Yonghong Zhou, Zhaobao Wang, Jianming Yang

**Affiliations:** 1 Energy-rich Compounds Production by Photosynthetic Carbon Fixation Research Center, Shandong Key Lab of Applied Mycology, Qingdao Agricultural University, No. 700 Changcheng Road, Chengyang District, Qingdao, 266109 China; 2 College of Life Sciences, Qingdao Agricultural University, Qingdao, China; 3 Institute of Chemical Industry of Forest Products, Key Laboratory of Biomass Energy and Material, CAF, Nanjing, China; 4 College of Food Science and Engineering, Qingdao Agricultural University, Qingdao, China; 5 Agricultural Integrated Service Center of Zhuyouguan, Longkou, Yantai, China

## Abstract

Plant biomass is a highly abundant renewable resource that can be converted into several types of high-value-added products, including chemicals, biofuels and advanced materials. In the last few decades, an increasing number of biomass species and processing techniques have been developed to enhance the application of plant biomass followed by the industrial application of some of the products, during which varied technologies have been successfully developed. In this review, we summarize the different sources of plant biomass, the evolving technologies for treating it, and the various products derived from plant biomass. Moreover, the challenges inherent in the valorization of plant biomass used in high-value-added products are also discussed. Overall, with the increased use of plant biomass, the development of treatment technologies, and the solution of the challenges raised during plant biomass valorization, the value-added products derived from plant biomass will become greater in number and more valuable.

## Background

Rapid population expansion and industrial development have led to a higher consumption of fossil fuels (coal, oil and natural gas) over the past several decades. Fossil fuels have relatively easy accessibility, compatibility and affordability [1], but they are non-renewable resources that will 1 day be exhausted. Excessive greenhouse gases (such as CO, CO_2_, NOx, SOx, and CH_4_) are discharged into the atmosphere following the consumption of fossil fuels [2], which has created a man-made climate change problem. Hence, environmentally friendly and renewable alternative energy sources have been explored, among which biomass energy is considered to be a clean energy source and a potential substitute for fossil fuels. As the world’s most productive biomass energy, bioethanol is basically produced from food crops rich in starch and sugar, which can cause an imbalance in the food and feed supply chain, challenging the sustainability of the process [3]. Lignocellulose biomass is a carbon-neutral renewable feedstock that is not edible and does not interfere with food and feed supplies [3].

Lignocellulose biomass consists of cellulose and hemicelluloses, lignin, and some other extractives. Cellulose and hemicellulose are polysaccharides that can be depolymerized into sugars, and then the sugars, which are used as platform molecules, can be converted into value-added products such as bioethanol, biobutanol, or itaconic acid through various biological pathways or chemical processing [4–6]. Lignin is composed of a variety of aromatic units, which can be extracted to produce aromatics or upgraded by tailoring microbes [7, 8]. Despite the high availability and low price of lignocellulose, there is strong resistance to its degradation, resulting in substantial challenges in using lignocellulosic biomass.

Therefore, it is necessary to remove, separate, and degrade the components of lignocellulose biomass through technical methods, and further treatments for different application purposes are needed to utilize plant biomass, in which the development and improvement of technical methods are of great significance for improving the processing procedures and utilization efficiency of plant biomass and for decreasing the accompanying adverse effects (such as environmental pollution and energy consumption). Great advances have now been made in lignocellulose biomass treatment methods, including pretreatment and lignin extraction. The aim of pretreatment is to disrupt the lignin structure and reduce the crystallinity of cellulose to increase enzyme accessibility, which is a necessary step for efficient enzymatic hydrolysis, because the physicochemical, structural, and compositional properties of lignocellulose biomass make it recalcitrant and difficult for enzymes to hydrolyse [9]. Lignin is a natural substance that is rich in aromatics and is usually produced in large quantities as a by-product of the paper industry. Lignin fractionation, lignin activation, and lignin depolymerization are performed through lignin extraction methods to achieve lignin valorization. However, some challenges have arisen during the development of cellulose utilization and aromatic monomer fractionation. Similarly, genetic engineering technologies have also been developed during the plant biomass utilization process. Overall, it is essential to explore and improve the corresponding methods and technologies for the utilization of massive lignocellulose biomass resources in the future.

This review summarizes the various types of lignocellulose biomass in common use, the technological development of lignocellulose biomass processing, and the types of value-added products derived from plant biomass. The challenges inherent to different treatment methods, lignin extraction and genetic engineering included in the valorization of plant biomass were also discussed.

## Categorizing generations of plant feedstocks

**Fig. 1:**
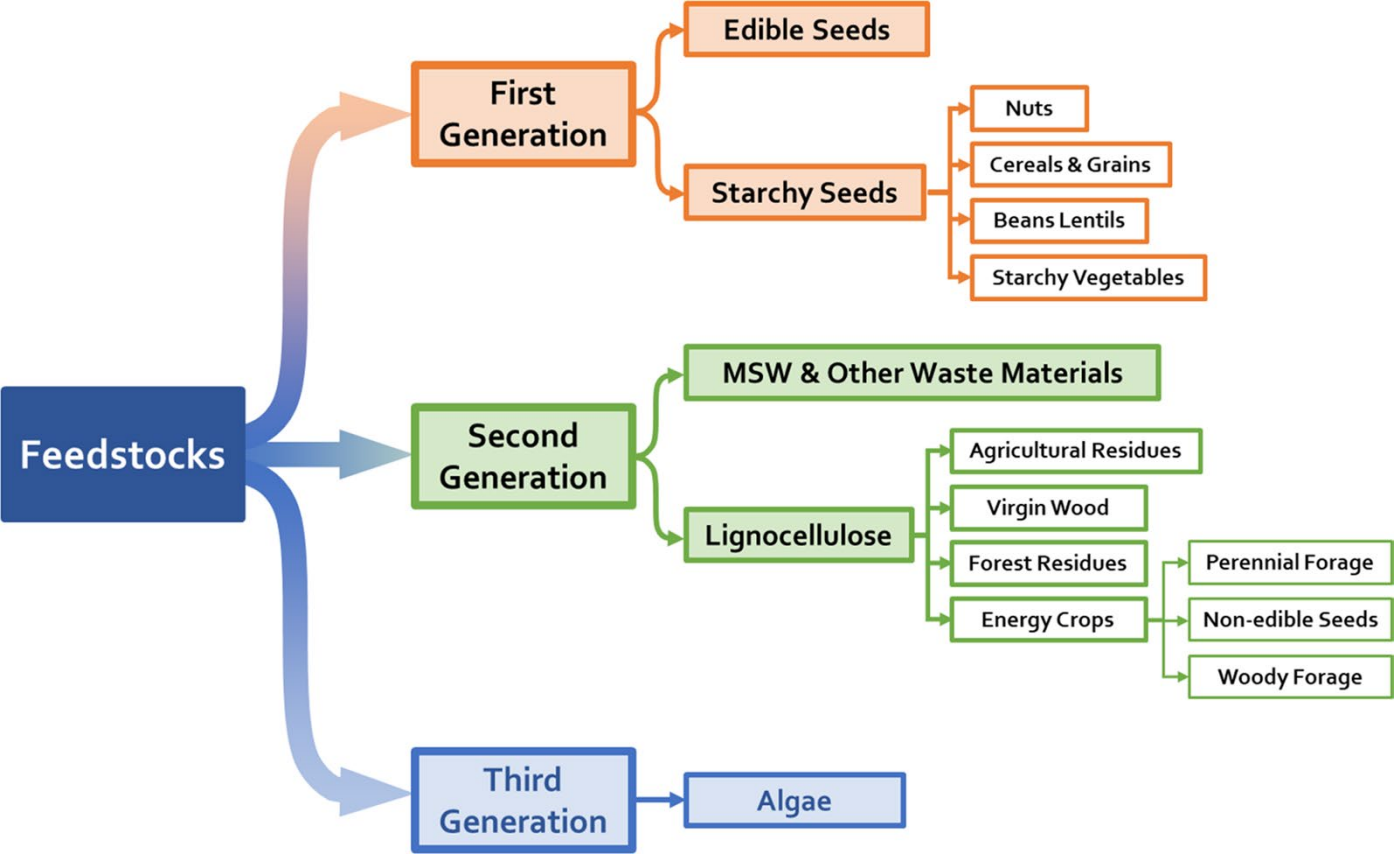
Taxonomy of commonly plant biomass resources used in valorization ( Adopted from Ref. [12])

In the context of the development of industry, the rapid increase in the population, the surge in energy demand and the intensification of the greenhouse effect, various types of biomass feedstocks (Fig. 1) have been discovered, and they are typically divided into three categories: first generation, second generation and third generation.

First generation feedstocks generally refer to plants that are rich in sugars (sugarcane, sweet sorghum, sugar beet, etc.), starches (corn, wheat, barley, potato, etc.) and oils (olive, palm, sunflower, coconut, etc.) and are used in the production of first-generation bioethanol and biodiesel. As the earliest researched biofuels, their industrial production techniques are already mature, and these biofuels have been used commercially in marine vessels or vehicles [10]. For example, the bioethanol used in the U.S. accounted for 46% of total biomass fuels in 2018 [11], and the global bioethanol production market reached 110 billion litres in the same year. However, first-generation feedstocks are primarily edible, so they will always compete with food crops or feed production. Furthermore, their production not only relies on fertilizers but also promotes deforestation to obtain more agricultural land [12], which makes first-generation biofuels non-sustainable. This is an incompatible and common theme of bioenergy; therefore, the second generation of sustainable feedstocks has been developed.

By contrast, second generation feedstocks primarily include non-food oil-rich plants (*Jatropha*, *Camelina*, rubber, jojobyl, kusum, and kapok) and lignocellulosic biomass (inedible crops such as *Miscanthus*, switchgrass, and reed canary grass; and agroindustrial and forest waste/residues). The sustainability of bioenergy is based on having sufficient land for production. Compared with first-generation feedstocks, cultivated areas are moving to marginal land for which the definition has been extended to describe degraded, abandoned, barren, idle, and underutilized lands, with the concept evolving [13]. For example, *Miscanthus* and switchgrass have a C_4_ photosynthesis pathway (nicotinamide adenine dinucleotide phosphate–malic enzyme), which is a high-efficiency photosynthetic mechanism for using nitrogen and water compared to that of C_3_ plant species. Moreover, *Miscanthus* and switchgrass also have low fertilizer demand and low susceptibility to diseases, allowing them to grow on marginal land. It has been suggested that planting energy crops on marginal lands with low vegetation coverage can reduce wind, degradation, water erosion, and CO_2_, sequester C, and improve the soil quality [13, 14]. The types of non-agricultural land and their potential availability for bioenergy production have been discussed [13]. Second-generation feedstock sources are abundant, such as mango peel, rambutan seed, acai seed, coffee residues (husk, pulp, and coffee cut-stems), peanut hull, and paper mulberry, which have been studied for producing biofuel or high-value compounds over the past few years [15–21]. However, the use of lignocellulosic biomass resources is still limited by less mature manufacturing technologies due to the demands for complicated pretreatment processes and high-cost equipment and reagents. Therefore, the development of cost-effective use processes has good research prospects.

Third generation feedstocks, primarily consisting of cyanobacteria and microalgae that are rich in lignocellulose, lipids and protein, are newly developed products. Algae has several advantages, including a fast growth rate, limited use of land, and high lipid production [22–24]. For example, microalgae can be harvested within 10 days after planting, and the productivity of biodiesel is 200 times that of conventional vegetables such as soybeans or rapeseed [25]. Currently, producing biofuels from third-generation feedstocks is considered to be the most promising way to meet the global energy demand [26]. Although this method has great potential, it is still in the early concept stage, and there are still many challenges to overcome, such as how to break algae cells and extract the required substances effectively [12].

## Characteristics of lignocellulosic biomass

Lignocellulosic biomass is the most promising renewable resource that could be used as a feedstock to produce biofuels and value-added compounds, which commonly consist of 10–25% lignin, 20–40% hemicellulose and 40–60% cellulose [27]. The contents of these three components of common plant feedstocks are listed in Table 1.

**Table 1:** Summary of the contents (wt %) of cellulose, hemicellulose and lignin in common plant feedstocks

Biomass	Cellulose	Hemicellulose	Lignin	Refs.	Biomass	Cellulose	Hemicellulose	Lignin	Refs.
*Crop residues*					*Hardwood*				
Rice straw	36.2–47	16–35	5.6–36.1	[181, 257, 258]	Hardwood stems	40–55	24–40	18–25	[14]
Rice husk	32.7–41.52	14.04–29.3	18.1–33.67	[258–261]	Poplar	42–49	16–23	21–29	[181]
Wheat straw	30–43.4	19.45–45.2	7.5–22.2	[258, 262–264]	Willow	36–39	21–22	19–20	[181]
Sorghum bagasse	27.3–45	13.1–36	14.3–25	[264, 265]	Eucalyptus	34.2–51.1	8.9–30.2	21.4–39.2	[266]
Sorghum straw	26.93	32.57	10.16	[264]	Oak	33.9–43.2	21.9–25.9	27.8–35.4	[264, 267]
Sugarcane bagasse	38.01–45	17.1–33.27	4.01–33.56	[143, 144, 258, 261]					
Corn stover	32–45	15.49–35	7–22.74	[14, 116, 262]					
Corn cob	39.3–52.49	23.7–35	12.5–19.6	[181, 261, 263]	*Softwood*				
Corn straw	51.53	30.88	17.59	[258, 263]	Softwood stems	45–50	25–35	25–35	[14]
Corn stalk	36.89	29.33	13.93	[261]	Beech	45.05–51.3	28–31.86	19.6–22.25	[261, 263, 268]
Tobacco residue	42.3–44.32	28.89–41.54	15.01–26.79	[263, 267]	Japanese larch	58.6	13.0	20.1	[269]
Barley straw	35.4	28.7	13.1	[264]	Pine	34–45.6	20.1–34.6	26–34.4	[181, 264]
*Wastes of fruit processing industries*					Spruce	24.7–47.11	10.2–21.31	31.58–35	[263, 264]
Banana peel	52.30	9.90	11.2	[267]					
Olive leaves	12.72–15.38	7.47–9.16	15.15–17.8	[270]					
Olive stone	30.10	17.10	32.6	[267]	*Grasses*				
Extracted olive pomace	40.00	22	19	[264]	Miscanthus	40–53	18–26.2	20–26.5	[14, 181, 271]
Acai seed	53.20	12.30	22.3	[17]	Switchgrass	39.5–45	20.3–31.5	12–20	[14, 181]
Cocoa shell	13.20	10.80	13.2	[267]	Bamboo	37–46.5	16.6–18.8	25.7–39.2	[264, 272]
Peanut shell	24.70	39.40	33.5	[267]	Agave bagasse	38.4–47.3	12.8–23.5	10.1–15	[273–275]
Walnut shell	40.10	20.70	18.2	[267]	Agave leaf	46–79.8	15.7–30	4.9–11	[275]

**Fig. 2:**
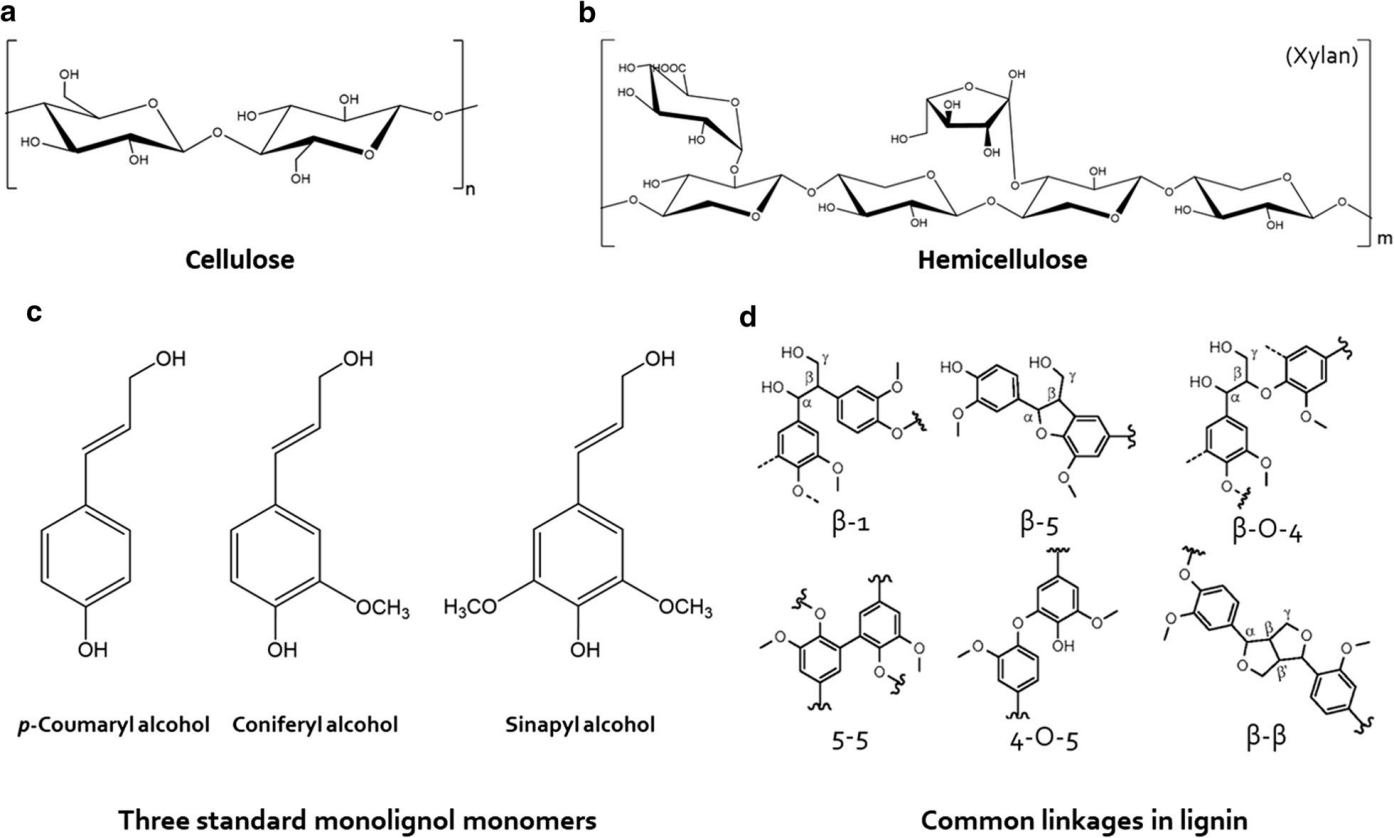
Structures of the main components involved in lignocellulosic biomass. **a** The partial chemical structural of cellulose; **b** The partial chemical structural of hemicellulose; **c** The structural characteristics of three typical units in lignin; **d** the common linkages between different lignin units ( Adopted from Ref. [34, 253])

Cellulose is a linear polymer with D-anhydroglucopyranose moieties in repeating units linked by β-(1-4) glycosidic bonds (Fig. 2a) [28]. In nature, approximately 20–300 cellulose chains are polymerized through hydrogen bonds and van der Waals forces to form cellulose fibres that are present in crystalline and amorphous forms [29]. As reported, cellulase has less of a degrading effect on the crystalline part of cellulose; therefore, crystalline cellulose degradability is 3–30 times lower than that of the amorphous form [30]. Nevertheless, cellulose still shows good potential for use after suitable treatments.

Hemicelluloses that are amorphous and random short-chain heteropolymers composed of two or more monosaccharides include pentoses (D-xylose and L-arabinose), hexoses (D-glucose, D-galactose, and D-galactose), and some low-content saccharides (L-rhamnose and L-fructose) (Fig. 2b) [31]. Furthermore, uronic acids and acetyl groups can also be found in hemicellulose; for example, arabinoglucuronic acids and glucuronic acids are components of softwood hemicellulose and hardwood hemicellulose, respectively [32]. Due to its own acetyl group and branched chain, hemicellulose lacks a crystal structure to make it easy to hydrolyse [33], but as a component of plant cell walls, hemicellulose interacts with cellulose and cross-linked lignin, which improves the mechanical strength of plants. Therefore, to release hemicellulose, pretreatment processes are required before further use, similar to that of cellulose.

Lignin plays a vital role in maintaining the structural integrity of plants and is primarily composed of three monolignols, *p*-coumaryl alcohol, coniferyl alcohol, and sinapyl alcohol (Fig. 2c). These monolignols make up three primary units (syringyl, guaiacyl, and hydroxyphenyl groups, which are abbreviated as S, G, and H, respectively), which have different numbers of methoxy groups (none, one, and two, respectively) connected to the aromatic ring [34]. As shown in Fig. 2d, the units are linked by carbon–oxygen (ether) bonds (β-O-4, α-O-4, and 4-O-5) and carbon–carbon (β-5, β-β, β-1, and 5-5) bonds [35–37]. The ratio of the three basic units varies according to the source of the lignocellulosic feedstocks, and the types and proportions of the linkages are also different. For instance, hardwood lignin is primarily composed of S and G units followed by traces of H units, and softwood lignin generally consists of G units with low levels of H units [38]. The C-3 and C-5 positions of sinapyl alcohol (S) are connected to the methoxy group, which cannot generate other intermonomer linkages with another unit; consequently, the S lignin structure primarily comprises β-O-4 bonds and small amounts of β–β [39]. By contrast, the C-5 of coniferyl alcohol (G) is unsubstituted, except β-O-4, and there are other linkages, mainly including β-5 and β–β as well as β-1 and 5–5, which are resistant linkages with higher degree of condensation [40]. As a result, softwood lignin has lower β-O-4 bond contents than hardwood lignin, so softwood lignin is relatively more difficult to use. Moreover, lignin is the most important obstacle to the use of lignocellulose. Almost all the studies on lignin degradation have focused on the destruction of the β-O-4 bond. However, the development of processes to destroy other linkages can more comprehensively provide for the valorization of plant biomass.

## Recent advances in lignocellulosic biomass treatment processes

**Fig. 3:**
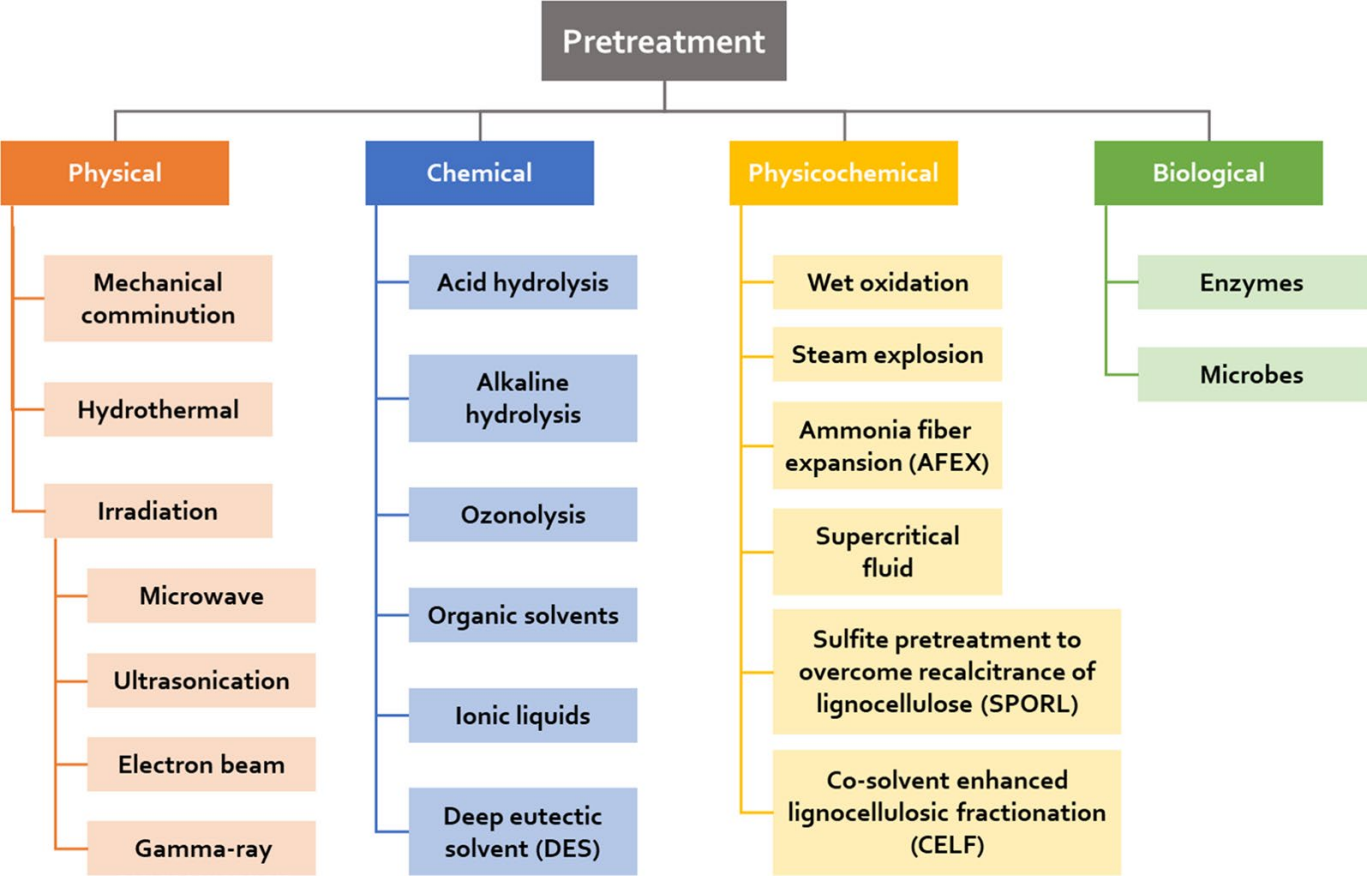
General taxonomy of pretreatment processes of plant biomass ( Adopted from Ref. [181, 254])

The mature conversion technologies developed with first-generation bioenergy production requirements are divided into two categories: thermochemical conversion and biochemical conversion. Torrefaction, pyrolysis, gasification and thermal liquefaction are commonly used in thermochemical conversion processes, which produce bio-char, bio-oil and syngas, while biochemical conversion processes include anaerobic digestion and fermentation. Lignocellulosic feedstock is recognized as a suitable feedstock for producing biofuels and value-added chemicals; however, its application within conversion technologies does not work well. For example, the rigid structure of plants will reduce the hydrolysis efficiency and directly affect methane production, which is estimated to be only approximately 50% [41]. As a complicated structure, a rigid structure is formed by lignin tightly cross-linked with cellulose and hemicellulose, which significantly limits enzyme contact with cellulose and hemicellulose, thereby reducing the release of sugar units. Therefore, research has focused on pretreatment processes to improve the biodegradability of plant biomass by removing lignin. Common pretreatment methods include mechanical comminution, acid, alkaline, organic solvent, ionic liquids, and steam explosion treatment, and all these methods are demonstrated in Fig. 3. Recently, a “lignin-first” process was proposed for extracting lignin from lignocellulosic biomass to produce aromatic compounds [34]. Some recent advances in lignocellulosic biomass treatment processes are summarized as follows.

### Recent advances in pretreatments

Deep eutectic solvent (DES), a mixture of hydrogen bond acceptors (HBAs) such as choline chloride and hydrogen bond donors (HBDs) such as amines, alcohols, lactic acid, and carboxylic acids, is a promising new green solvent that can be used as an alternative to ionic liquids, and the typical DES is mixed with choline chloride (ChCl) and lactic acid (LA) [42]. DESs have similar physical and chemical properties compared to ionic liquids [43], but they show a lower melting point, greater stability and lower cost [44]. Additionally, there are some advantages of DESs for the following treatment, including low toxicity and biocompatibility. Moreover, the barely volatile components of DESs make them easy to recycle and reuse with no influence on the treatment efficiency [45, 46]. DES pretreatment demonstrates specific selectivity to lignin, resulting in high-purity lignin extraction, which favours the fractionation and valorization of lignocellulose [46]. Therefore, DES has become the focus of recent research, which showed some novel processes based on DES and the characteristics of DES pretreatment. An appropriate hydrogen bond can be formed between the Lewis acid and DES, which significantly facilitates the degradation of lignin and hemicellulose. In a three-phase pretreatment system composed of FeCl_3_ and chloride/glycerol DES, 93.63 wt% hemicellulose and 78.88 wt% lignin from *Pennisetum* were removed, while 95.2% cellulose was retained [47]. Additionally, AlCl_3_ performs a similar function in a DES system that uses lignin-derived guaiacol as the HBD [48]. However, during the washing process after the pretreatment, the degraded lignin precipitates easily, thereby inhibiting enzymatic hydrolysis in the next step. Several studies have shown that this problem can be solved by alkali washing, which can remove the precipitated lignin, followed by the conversion of near complete cellulose [48, 49]. Furthermore, the characteristics of DESs, including the relationship between the physicochemical properties of HBD, the effect of ChCl-based DES treatment and the chemical modification of hemicellulose and lignin during ChCl/LA DES treatment, are discussed, and it has important guiding significance for understanding the mechanism of DES pretreatment [50, 51]. Nevertheless, although DES is the focus of the pretreatment, this process is still immature, and more efforts should be made to reveal its mechanism of action.

Steam explosion (SE) is considered an effective and economical physicochemical pretreatment technology for the industrial scale [52], and it only uses water without any catalyst [53]. In this pretreatment, high temperature and pressure (20–50 bar, 160–290 °C) are maintained for a period of time, and then the process is terminated by sudden decompression to atmospheric pressure [54]. When pressure is swiftly released, the steam molecules that have leaked into the plant biomass are released instantaneously. The energy in the steam is converted into mechanical energy that acts on lignocellulose, resulting in some hemicellulose solubilization and hydrolysis and lignin transformation and partial removal [53, 55]. In addition, during the treatment, organic acids such as acetic acid are formed from acetyl or other functional groups on lignocellulose, and water can also be regarded as an acid at high temperatures, and it further catalyses the hydrolysis of hemicellulose [56]. After SE pretreatment, a large amount of oligosaccharides can be obtained, while holes with diameters within 10–20 nm will be effectively generated in the feedstocks [57], which is beneficial for subsequent enzymatic hydrolysis or extraction. Under optimal SE pretreatment conditions, 71.62% xylan, 29.47% glucan and 22.21% arabinan were extracted from industrial vinegar residue, and in the subsequent enzymatic hydrolysis process, the hydrolysis rate increased 13-fold compared with that without pretreatment [57]. Similar results were obtained with hybrid *Miscanthus*, for 52% xylo-oligosaccharides (XOS), and the production of fermentable sugars can be improved by 8~9-fold [58]. As the SE pretreatment matured, some improved processes based on SE pretreatment were explored, including ammonia fibre explosion (AFEX) and dry explosion. Compared with the SE pretreatment, AFEX replaces water with ammonia, the boiling point of which is lower than that of water. As the chemical treatment part of AFEX, ammonia can affect lignocellulose, along with the breakdown of the carbohydrate linkages with lignin and the partial decrystallization of cellulose [55]. As reported, after AFEX treatment, the fibre crystallinity of corn distillers dried grains with solubles (DDGS) can be reduced to 0%, increasing the digestibility and fermentability of corn DDGS *in vitro* [59]. It should be noted that ammonia treatment can reduce the solubility of cellulose and crystalline lignin and has a poorer effect on lignin-rich and woody feedstocks [60]. Moreover, dry explosion pretreatment showed lower energy consumption than SE under the same temperature conditions and enhanced the fuel properties of the biomass [61].

A novel pretreatment method, SPORL (Sulfite pretreatment to overcome recalcitrance of lignocellulose), has been established for a more stable and effective bioconversion of softwood lignocellulose. In this technology, wood feedstocks were pretreated in an aqueous solution of sulfite and/or bisulfite [62]. During SPORL treatment, the recalcitrance of wood material can be reduced via combined effects, including partial delignification, the dissolution of hemicelluloses, increased surface area, the depolymerization of cellulose, and the partial sulfonation of lignin [63]. Furthermore, SPORL can dissolve partial lignin and almost all hemicelluloses through lignin sulfonation [64].

There are various common challenges during the pretreatment process, such as the condensation of dissolved lignin, sugar degradation products, and difficult solvent recovery. To solve these problems, OrganoCat was recently proposed, which is similar to Organosolv but employs a biphasic solvent system (oxalic acid and 2-methyltetrahydrofuran); nevertheless, it was developed based on mature acid pretreatment [65]. In other words, OrganoCat is an improvement of the mature acid pretreatment method, in which oxalic acid and 2-methyltetrahydrofuran (2-MeTHF) are applied as a catalyst and an extractive phase for hydrolysing amorphous hemicellulose and extracting lignin in situ from the reactive phase, respectively, resulting in three produce streams, namely cellulose-rich solid pulp, organic phase-containing lignin, and acid-containing aqueous phase-containing hemicellulose [66, 67]. To date, this technology has been studied on several different types of biomass feedstocks, but the treatment effect on each biomass feedstock varies greatly. For instance, rice straw seemed to be well suited for OrganoCat, which was accompanied by 98.99% cellulose recovery, 88.79% hemicellulose solubilization, and 71.46% lignin removal after OrganoCat treatment [68]. By contrast, OrganoCat does not have much effect on eucalyptus, and only low amounts of lignin and sugars can be extracted [66]. As a method designed for refinery applications, OrganoCat has some promising characteristics, such as easy and effective fractioning, fewer sugar degradation products, and more efficient recovery efficiency, which result in minimized energy and mass consumption [69]. To understand the valorization of lignocellulosic feedstocks, some research has addressed the valorization of lignin extracted by the OrganoCat process, which is discussed in section 4.2.

An acid-free and mildly oxidative organosolv delignification process, the novel “OxiOrganosolv” pretreatment method, was recently reported [70]; this method is an improvement of the acetone/water oxidation (AWO) process that was developed on the basis of the wet oxidation pretreatment method by replacing water with an acetone/water mixture. Compared to steam explosion, the AWO process not only maintains the advantages of typical wet oxidation (fewer degradation products and low temperature) but also achieves higher biomass delignification [71]; based on these advantages, OxiOrganosolv further improves the efficiency of delignification. The biomass and organic solvent/water mixture with a solid–liquid ratio of 1:10 are added to an autoclave reactor pressurized with 100% O_2_, and an OxiOrganosolv treatment is performed at a specific temperature and time. Then, the liquid fraction containing water, dissolved lignin, dissolved lignin and dissolved lignin is separated from the solid residue containing cellulose by vacuum filtration [70]. This research showed that cellulose recovery and the degree of delignification with solvents (acetone, ethanol and tetrahydrofuran) can reach 100% and 95%, respectively, during the pretreatment of beechwood and pine material under optimal conditions. It is worth noting that the efficient single-stage organosolv delignification of softwood has not succeeded when using other organosolv variants in the past. However, as a new method, although OxiOrganosolv has many advantages, more research on the usen of fractionated lignin and hemicellulose must still be developed, and the economic viability of the application should be explored.

Notably, the basic pretreatment methods used at the beginning, such as mechanical comminution, irradiation, and acid and alkaline hydrolysis, are now mostly used as assisted or preliminary treatment methods over the entire pretreatment process [72, 73]. Additionally, some studies combine two or more pretreatment methods to compensate for the shortcomings associated with a single method or to increase the production of target substances [74, 75]. Furthermore, more pretreatment methods or new methods derived from mature methods are needed to achieve fewer shortcomings and higher economic benefits.

### Recent advances in lignin extraction

After pretreatment, most of the cellulose is extracted, leaving the residue primarily consisting of lignin, which would be used as waste to burn, to supply heat and energy [76]. However, lignin is a natural substance that contains a large count of aromatic units, and the annual production of lignin can reach approximately 5×10^6^ metric tons [77], which makes it a potential feedstock for producing aromatic-related value-added products such as aromatics, phenolics, biofuels, and macromolecules [35, 78]. In nature, among the linkages, β-O-4 is the most abundant linkage in native lignin [79], accounting for approximately 50% of the lignin in softwood and greater than 60% in hardwood [46]. Therefore, the method of breaking an appropriate amount of β-O-4 bonds has become an important aspect of lignin extraction. Over the past few decades, efficient strategies for lignin depolymerization have been explored, including alkali-based, acid-based, reductive-catalytic, oxidative-catalytic, pyrolysis, photocatalytic depolymerization, and enzymatic hydrolysis treatments, all of which are described in detail as follows.

Photocatalytic lignin depolymerization has attracted increasing attention in recent years because of its mild reaction conditions, simple reaction process, and great environmental friendliness [80, 81], making it a potential method for replacing the traditional process of lignin depolymerization. The electrons in the photocatalyst are brought into their excited state by light, which may substitute reductants, and each electron transition will produce a hole that may substitute oxidants [82, 83]. Hence, the C–O bonds in β-O-4 can be cleaved by photocatalysis via three pathways: oxidative cleavage promoted by holes or oxidative species, reductive cleavage initiated by electrons or reductive agents, and redox neutral cleavage as accomplished by electrons combined with holes/oxidative species [84]. For example, in a two-step photocatalytic strategy, C_α_–OH is aerobically oxidized to C_α_=O using a Pd/ZnIn_2_S_4_ catalyst under λ=455 nm light irradiation, and then the C–O bond is cleaved by TiO_2_ under λ=365 nm light irradiation [85]. While this is an overall one-step redox-neutral reaction, sacrificial agents are required to consume the unused holes or electrons at each step, which still generates a great deal of stoichiometric waste [85, 86]. In addition, the catalyst activity and the cost of expensive reagents and catalysts remain to be improved and addressed, respectively, which restricts the application of photocatalysis [82, 86].

Recently, co-solvent enhanced lignocellulosic fractionation (CELF), a “lignin-first” strategy, has been developed for the efficient fractionation of lignin from lignocellulose feedstocks, in which tetrahydrofuran, which can dissolve acetylated lignin, is used as a co-solvent with water under acidic conditions [87, 88]. In CELF, a high temperature (>150 °C) is still needed to remain outside the known miscibility gap (71.8 °C to 137.1 °C) [89] of the THF(tetrahydrofuran)/H_2_O mixture. Generally, approximately 85–90% lignin from plant biomass can be solubilized and fractionated, because the molecular weight of lignin is dramatically reduced and the cross-condensation reaction is also minimized under THF/H_2_O mixture treatment [90]. The nearly pure lignin product without sugar and ash can be precipitated after THF solvent evaporation, which is a potential feedstock for promoting the hydrolysis of cellulose [91]. Almost complete glucose recovery (>99%) could be achieved after the enzymatic hydrolysis of corn stover when pretreating with CELF [87]. Some research suggests that several solvents, such as γ-butyrolactone, γ-valerolactone, dimethyl sulfoxide, 1,4-dioxane, and acetone, can also be co-solvents for biomass delignification in CELF [92, 93]. Moreover, CELF could also be applied to produce fuel precursors, including furfural, 5-hydroxymethylfurfural, and levulinic acid [94].

In addition, during the OrganoCat process, lignin can be extracted in situ from the reactive phase by the 2-MeTHF phase, therefore, maintaining the low concentration and structural integrity of lignin and reducing the occurrence of condensation reactions while retaining a higher number of aryl ether linkages, which is more beneficial for lignin valorization [66]. Surprisingly, the monomer composition of lignin from different lignocellulosic feedstocks showed different changes after the OrganoCat process, among which those from beech wood and *Miscanthus* were not changed. In addition, the S/G ratio increased for *Sida*, consistent with previous results [95]. Until the antisolvent precipitation method was reported last year, there was no other suitable process for using the extracted lignin produced in the OrganoCat process. As reported, antisolvent precipitation adopting n-pentane combined with solvent evaporation and filtration achieved a 70% lignin precipitation yield and the almost complete recovery of *n*-pentane [96]. Hence, this OrganoCat process may consume minimal energy and minimal antisolvent amounts in the separation of lignin.

The lignin fractionated by most treatment methods is modified and condensed lignin, which is very different from natural lignin and is not suitable for further valorization. Then, enzyme-assisted extraction was developed to obtain lignin, which primarily uses cellulase to remove carbohydrates from milled lignocellulose materials, resulting in improved yields of cellulolytic enzyme lignin (CEL) [76]. A novel method has been developed by combining enzymatic hydrolysis and alkaline treatment, in which the ball-milled swollen cell wall is first treated with a mild alkali, followed by enzymatic hydrolysis, leading to a 95% yield of swollen residual enzyme lignin [97]. However, although enzymatic hydrolysis can reduce the use of toxic organic reagents, the treatment time increased.

## Products derived from plant biomass

**Fig. 4:**
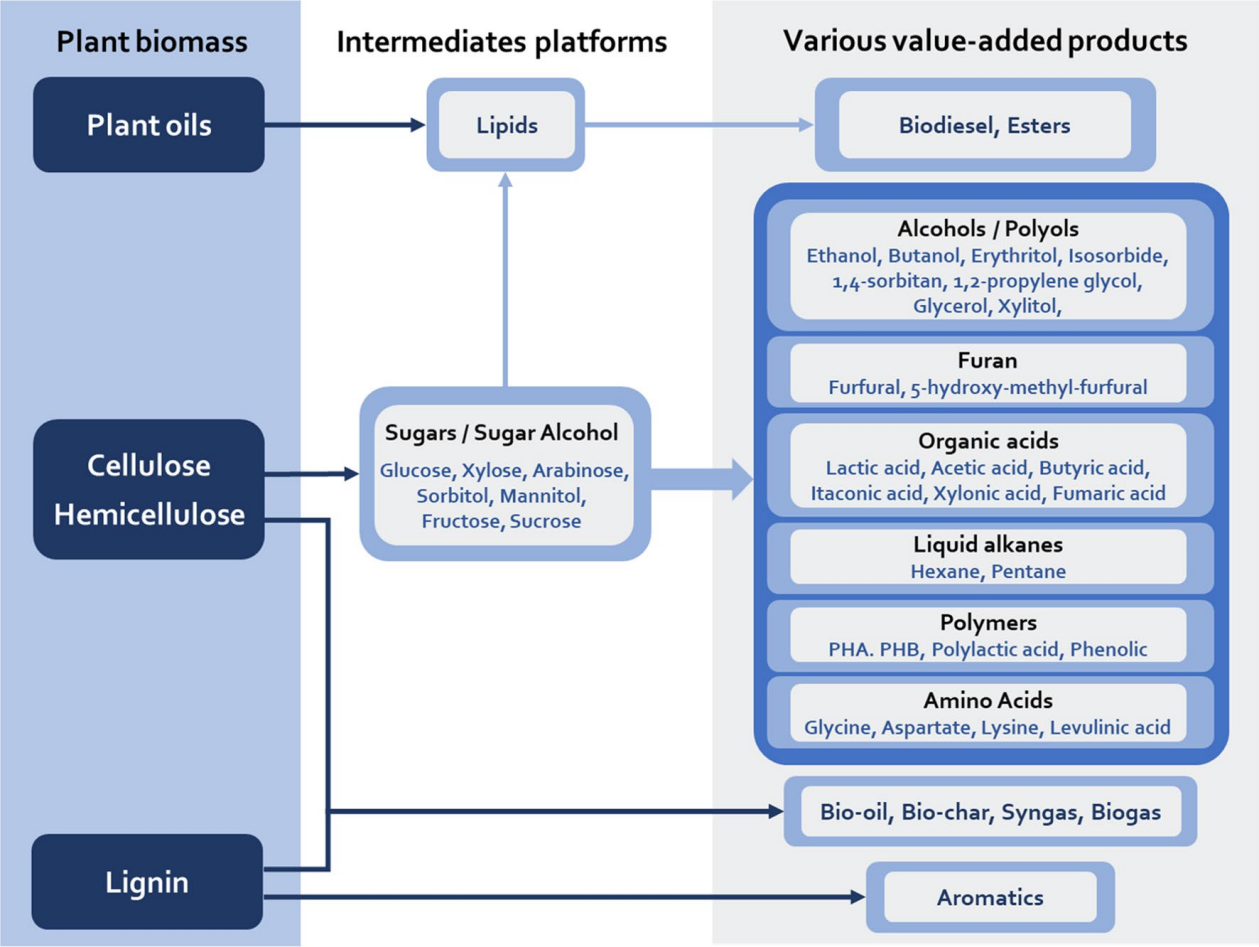
Summary of the various products derived from plant biomass

The diverse target products derived from lignocellulose feedstock can primarily be divided into three categories: monosaccharide and sugar alcohol; biodiesel; bio-oil, bio-char, and syngas, whose downstream products, such as alcohols, diols, carboxylic acids, organic acids, polymers, furfural, bio-gas, liquid alkanes and phenol [3], show diverse applications in different industries, such as pharmaceuticals, biomedical products, agrochemicals, aerospace, the building sector, filler materials, fragrances, food, cosmetics, etc. [98–102] All the mentioned products derived from plant biomass are listed in Fig. 4.

Lignocellulose biomass is very rich in pentose and hexose sugars. After pretreatment and enzymatic hydrolysis, monosaccharides, sugar-related alcohols and acids are obtained, such as glucose, xylose, arabinose, sorbitol, mannitol, glucuronic acid, and galacturonic acid [103]. Among these compounds, sorbitol, mannitol, glucose, and xylose then become the primary carbon sources for the next step. Sorbitol and mannitol can be used as the precursor compounds of isosorbide, lactic acid, and 1,4-sorbitan [104–106]. Isosorbide is a commercial platform chemical that is usually used as a precursor to synthesize isosorbide-based polymers, such as poly(arylene ether ketone)s, a type of super-engineered plastic [107], and some polymeric nanofibres that can be used in industrial applications [108]. In addition, isosorbide can be further converted into hexanol, hexane, and pentane [109]. Recently, several methods have been proposed that could be used to directly produce isosorbide from lignocellulose or cellulose biomass [110, 111]. Glucose can be converted into 1,2-propylene glycol by hydrogenolysis, which can selectively cleave the C–C and C–O bonds of glucose [112] or hexane via hydrodeoxygenation [109], and glucose can also be fermented by microorganisms into ethanol, erythritol [113, 114], lactic acid [115], organic acid [116, 117], polyhydroxyalkanoate (PHA) [118, 119], and polyhydroxybutyrate (PHB) [120]. In addition, glucose can also be fermented into furfural, which, as an inhibitor of the next fermentation, can be produced during the pretreatment of biomass feedstocks, and furfural is also a platform compound for fuel additives, medical drugs, and other bulk chemicals [121–123]. Xylose, the primary component of hemicellulose, can be fermented into ethanol, isobutanol, xylitol, xylonic acid, 3-hydroxypropionic acid, PHB, etc. by engineered yeast strains and *Corynebacterium glutamicum* [124–129]. In addition, glucose and xylose can be converted into ethanol/butanol/acetone, succinic acid/itaconic acid, and fumaric acid by *Clostridium*, *Actinobacillus succinogenes* (DSM 22257), and *Rhizopus arrhizus RH 7-13-9#*, respectively [130, 131].

Biodiesel is a renewable alternative to fossil fuels [132] that is usually produced from vegetable oils and animal fats using alcohol (generally methanol or ethanol) and catalysts (generally NaOH or KOH) through a transesterification process [133]. During this process, crude glycerol is produced as a by-product, which accounts for almost 10% of the total end-products [134–137]. However, there is no economic benefit of further purification. Researchers have found that crude glycerol can be processed to produce high-value products such as polyols and polyurethane foams by chemical treatment [138], and ethanol, butanol, propionic acid, citric acid, xylitol, erythritol, etc. can be produced by fermentation [139–142]. Moreover, crude glycerol can be reused in the pretreatment of bagasse to enhance L-glucose production [143, 144], improve the treatment effect and increase the subsequent saccharification efficiency. Recently, the strategy of glycerol-free biodiesel production has been developed [1]. Furthermore, to reduce the use of vegetable oil as biodiesel feedstock to decrease the competition between food and fuel, strategies have been proposed in which microbial lipids could be produced as biodiesel feedstocks through biological pretreatments of empty fruit bunches or lignin-containing paper industry wastewater by oleaginous microorganisms [145, 146].

Bio-oil is another alternative to conventional fuels; however, some of its shortcomings, such as high viscosity, high water content, high oxygen content, chemical instability and corrosiveness, limit its applications [147–149], and its issues can be addressed by fast pyrolysis or other methods [150–152]. Acids, alcohols, furans, and phenols, etc. have been detected in bio-oils under most conditions, among which phenols are dominant [150], and phenols can be used for phenolic resin production or other applications [153]. Additionally, pyrolysis is also commonly used to produce bio-char and syngas. Bio-char has received more attention in recent years, and several reviews have summarized its production [101, 154–157]. Bio-char presents porous structure, abundant functional groups, high surface area, which includes minerals and trace metals [158], so it shows a wide range of applications, including adsorption (chemical substances [159, 160], dye [161, 162], and heavy metals [17, 163, 164]), catalysis [165–168], soil repair [163, 169], and use as building materials [170], electrochemical energy storage [171–173], etc. During the process of bio-oil or bio-char production, syngas containing hydrogen, carbon monoxide, methane, etc. is usually performed as a by-product [151], which can be directly combusted to generate electricity or heat and converted into high-value products such as methanol or dimethyl ether [174, 175]. In addition, gasification and microbial anaerobic fermentation can also produce bio-gas, primarily hydrogen and methane [176–179], and methane can also be used in the improved pyrolysis of upgraded bio-oil production [150].

To reduce the adverse effects of excessive fossil fuel consumption, biomass fuel is regarded as a green and renewable alternative to fossil fuels, which has attracted widespread attention. Among biomass fuels, bioethanol is the most studied and the primary industrial application. More than 96% of the total global bioethanol production still comes from the first-generation bioethanol (1G) produced from grains and starch-based feedstocks such as sugarcane, sugar beet and corn; moreover, these feedstocks are not considered sustainable in the long term due to their direct or indirect competition with food and feed production [180]. By contrast, the second-generation bioethanol (2G) produced from lignocellulosic biomass, such as woody crops, crop residues or energy grasses, can not only address the problem of food competition but they also demonstrate a higher potential to reduce the greenhouse effect [181–183]. If 2G fuel reaches biorefinery level applications, many problems, including technical and economic problems in the biochemical transformation route and local biological resource collection and processing and life cycle assessment, must be solved and overcome in the future. Recently, some attention has been given to the production of biobutanol, because bioethanol has some obvious shortcomings as a substitute for gasoline. Ethanol has great differences in hygroscopicity and specific combustion energy density, resulting in a mixture ratio of ethanol and gasoline creating a challenge for motor operations. By contrast, butanol shows no hygroscopicity and can be easily added to gasoline in any proportion [184]. However, the relevant research at this stage is largely focused on the metabolism and genetic engineering of *Clostridia* strains used for butanol fermentation and the optimization and innovation of fermentation conditions [185–189].

## Challenges in the valorization of plant biomass

As reviewed, great advances have been made in the valorization of plant biomass in recent years, including in various feedstocks, treatment methods, and target products. However, some challenges have arisen during the development of cellulose utilization and aromatic monomer fractionation. In fact, the challenges raised during the two processes mostly involve the lignin component. The tight structure of lignin is a barrier to prevent macromolecular enzymes such as cellulase from passing through and binding to cellulase non-productively, which limits enzyme contact with cellulose and hemicellulose [190], resulting in a serious effect on the enzymatic hydrolysis efficiency of cellulose. During the fractionation of aromatic monomers, whether under acidic or alkaline conditions, the benzyl group generated by the cleavage of lignin is subsequently attacked by the nucleophile to form stable and condensed C–C or C–O bonds, which significantly limits the completion of lignin valorization [191, 192]. Here, the challenges in the use of cellulose and the fractionation of aromatic monomers were summarized. Similarly, applied genetic engineering technologies were also discussed.

### Challenges in using cellulose

Lignocellulosic material is one of the largest biomasses produced in the world and has received great attention in association with the urgent need for biofuels. However, the presence of lignin in lignocellulose feedstocks seriously affects the efficiency of sugar production, which is a platform compound for producing value-added products. Various pretreatment methods have been proposed to remove lignin or increase the exposure of cellulose, each of which shows their respective advantages and disadvantages, as shown in Table 2. In addition, all the types of biomass raw materials differ in their cellulose, hemicellulose, lignin, water, oxygen contents, etc., which will directly influence the efficiency of each pretreatment method under different conditions. Therefore, the pretreatment should be meticulously selected depending on the various characteristic properties of different lignocellulose biomass materials.

**Table 2:** Advantages and disadvantages of lignocellulose biomass pretreatment methods. ( Adopted from Refs. [14, 33, 255, 256])

Category	Pretreatment	Advantages	Disadvantages
Physical	Milling	Control of final particle size Reduces cellulose crystallinity Cost-effective especially for agricultural residues	High consumption of power and energy High energy required for hardwood biomass
	Steam explosion	Cost-effective for hardwood High concentrated sugars Lignin transformation and hemicellulose solubilization Low capital investment, moderate energy requirements and low environmental impacts	Hemicellulose is partly degraded Sugar degradation might happen Less effective for softwood Efficiency is affected by particle size
	Liquid hot water	Enhance cellulose digestibility, sugar extraction, and pentose recovery, No need for additional acid and size reduction low-cost reactors low or no inhibitor production	Water and energy demanding are higher
	Microwave	Less reaction time, Selectively heats for polar part Low inhibitor production	High cost Low effective for materials with low dielectric loss factor
	Ultrasonication	No external reagents are needed	Increase of cost for larger scales
Chemical	Acid hydrolysis	Hemicellulose and partly lignin are removed High reaction rate	Corrosion problem of reactor. High inhibitory formation from sugars degradation Requirement of neutralization
	Alkaline hydrolysis	Decrease in the polymerization of carbohydrates Efficient removal of lignin Low inhibitor formation Low temperature and pressure	Relatively long reaction time Low digestibility enhancement in softwood Requires alkali removal High cost of alkaline catalyst
	Ozonolysis	Reduces lignin content Low inhibitor formation Room temperature and atmospheric pressure	High cost of large amount of ozone needed Flammability and toxicity
	Organic solvents	Solubilization of lignin and hemicellulose Pure cellulose yield High glucose yield Lignin recovery	High cost of energy and catalysts Inhibitor generation Fire and explosion hazard Recycling of solvent and/or catalysts.
	Ionic liquids	Mild reaction conditions Requires no catalyst and low-cost reactor Ionic liquids are recyclable and reusable Lignin extraction can be achieved	Toxicity and inhibitory effects on enzyme activity High ionic liquids costs Requirement of ionic liquids recovery.
	Deep eutectic solvent (DES)	Green solvent, biodegradable and biocompatible High-purity lignin	Poor stability under higher pretreatment temperatures
Physicochemical	Wet oxidation	Efficient removal of lignin Low formation of inhibitors Reduced crystallinity of cellulose	High cost of oxygen Cellulose degradation High cost of corrosive resistant reactor Low hemicellulose recovery
	Ammonia fiber expansion (AFEX)	Cellulose crystallinity can be reduced Short reaction time High efficiency and selectivity for lignin Lower inhibition	Requires ammonia recycling system Less effective for softwood High cost of large amount of ammonia Environmental concerns
	Supercritical fluid	High solid load Low sugar degradation Output controllable by some factors Increases accessible surface area	High costs of energy consumption and reactor High pressure requirement
	Sulfite pretreatment to overcome recalcitrance of lignocellulose (SPORL)	Effective for hardwood and softwood Cost efficient Low inhibitor	Pretreatment is preceded by biomass size reduction
	Co-solvent enhanced lignocellulosic fractionation (CELF)	Highly efficient for lignin extraction Nearly pure lignin production	High cost of solvents High temperature requirement
Biological	Enzymes	Mild reaction conditions Environment friendly Selective degradation of lignin	Very long reaction time Low hydrolysis rate High environmental requirements Inactivate easily High cost of enzymes
	Microbes	Have better tolerance for the environment than enzyme	Long pretreatment time Requires careful control of growth conditions

To date, most of the studies on plant biomass utilization have been performed at the laboratory scale. For industrial application or commercialization, the experimental scale must be expanded to identify and address the challenges encountered in the commercialization of biomass pretreatment. Physical pretreatment is considered to be the most available method for large-scale implementation, because treatment systems are limited in cost and/or complexity [193]. Although chemical pretreatment has been studied at the bench scale [194], it is not suitable for use at a large scale, because some chemical reagents are expensive, and corrosivity increases the requirements for equipment. In addition, the need for solvent recovery, inhibitor removal and pH control increases the complexity of the chemical treatment system. If the solvent cannot be recovered, it would not only increase the cost, including the need for the subsequent treatment of used solvents and large amounts of new solvents but also cause environmental pollution. Therefore, green solvents, which can reduce impacts on health and the environment, have been developed for lignocellulose biomass pretreatment. Nevertheless, this strategy is still incipient, and many issues, such as process optimization, life cycle analysis, green solvent separation and recovery, must be addressed [195, 196]. Recently, biological pretreatment has been considered to be another environmentally friendly method that has many advantages, such as mild reaction conditions without high temperature and high pressure treatment or special equipment, no environmentally hazardous substances, and basically no inhibitors. By contrast, the cost and time consumed in biological pretreatment have become major issues. When lignin-degrading enzymes were used for pretreatment, the structural complexity of different lignocellulosic feedstocks would affect the combination of enzymes, affecting the efficiency of enzymatic hydrolysis and the time of the treatment process. Otherwise, the enzymes used in the process cannot be recycled, which significantly increases the cost. Furthermore, wild or genetically engineered fungi or bacteria that can produce lignin-degrading enzymes have been applied for lignin depolymerization. The exploration of microorganisms that can naturally depolymerize lignin and genetic engineering technologies enabling microorganisms to overexpress lignin-degrading enzymes have become one of the research directions in the biomass utilization field [197–199].

### Challenges in the fractionation of aromatic monomer

The large molecular weight and heterogeneous structure of lignin will seriously affect its potential applications in the materials field [200]. Although natural lignin is highly reactive towards depolymerization, during the biorefining process, severe structural degradation often occurs, including the cleavage of unstable ether and ester bonds (primarily β-O-4 ether bonds) in which the benzyl group is formed, which is the source of the lignin condensation reaction [79, 201]; this reaction is the primary factor affecting the formation of stable intermediates and maximizing the yield of phenolic monomers. Moreover, the excessive structural modification of the extracted lignin would affect its high-value applications. To reduce the influence of these factors, some improved methods or strategies have been proposed as follows.

#### Chemical structure modification of the lignin

Acid is the most commonly used catalyst for extracting aromatic monomers from lignocellulose biomass. However, during the acidolysis process, the C_α_^+^ carbocation formed by the dehydration of C_α_H-OH of β-O-4 could attack the adjacent aromatic rings with G or S structures and form new C–C bonds [202, 203], which is the condensation of lignin. Therefore, some methods have been proposed to reduce or avoid lignin condensation, including lignin pre-oxidation to prevent the formation of β-O-4 carbocations and the addition of capping reagents to block the reactive sites to stabilize the resulting β-O-4 carbocations [8], such as the stabilization of the monomer through acetal formation.

Pre-oxidation, a strategy proposed earlier, has been extensively studied in recent years. The pre-oxidation mechanism is to oxidize the C_α_-OH in the β-O-4 structure to C_α_=O selectively before lignin depolymerization, which not only inhibits repolymerization by avoiding the generation of carbocations but also significantly decreases the bond dissociation energy (BDE) value of the β-O-4 bond [8, 204]. Therefore, pre-oxidized lignin can be depolymerized under mild conditions. For example, oxidized poplar lignin is treated in a formic acid solution at 110 °C to obtain a yield of 52.2 wt % phenolic monomers, which is more than 7 times higher than that of unoxidized poplar lignin [205]. In addition, several oxidation methods have been improved, including stoichiometric, metal-free catalytic and metal-catalysed aerobic oxidation [206], even though the oxidation process can be promoted by photocatalysis [8]. It should also be noted that the BDE of the C_α_–C_β_ bond would increase after pre-oxidation [207]; as a result, pre-oxidation may not be suitable for treatments that require the subsequent cleavage of the C_α_–C_β_ bond.

During the lignin extraction process, the acetal formation of monomers can prevent the cleavage of the β-O-4 ether bonds and lignin condensation, thereby improving the efficiency of monomer production [201]. In 2016, a novel strategy was created to inhibit the lignin condensation reaction by forming a 1,3-dioxane acetal structure after adding formaldehyde as a protecting reagent [208]. Subsequently, the properties of acetaldehyde and propionaldehyde were studied, and it was found that the yield of phenolic monomers produced with formaldehyde was the highest (46 wt %), followed by those produced with propionaldehyde (42 wt %) and acetaldehyde (37 wt %), respectively [209]. Furthermore, the stabilization of lignin C2-aldehydes can be achieved through acetal formation with the addition of ethylene glycol (EG), which can be readily dissolved in organic solvents and catalyse depolymerization [210]. Recently, a mild hydrogenolysis method of H_2_SO_4_ with dimethyl carbonate as the solvent and ethylene glycol as the stabilizer was developed, resulting in a C2-acetal phenolic monomer yield of 77–98% [211].

Over the past 5 years, in situ lignin modification has been proposed for acid and alkali pretreatment and has been shown to be an effective method for eliminating the inhibitory effect of residual lignin. Carbocation scavengers such as 2-naphthol, 2-naphthol-7-sulfonate and vanillic acid have been added during acid pretreatment to prevent lignin repolymerization [212–214]. Additionally, during alkali pretreatment, lignin is modified in situ with PEGDE, which not only chemically blocks some phenolic hydroxyl groups in lignin but also increases the lignin hydrophilicity [191]. The addition of 2-naphthol could reportedly increase the delignification ratios from 16.6% to 18.2% during the acid pretreatment of larch with 1% (w/w) sulfuric acid at 160 °C for 1 h [215]. Although the in-situ modification is not specific for lignin extraction, it still provides a theoretical reference for improving the yield of aromatic monomers.

Additionally, due to the higher amount and lower bond dissociation energy of the C–O–C ether bond in comparison with the C–C bond, the cleavage of the C–O–C ether bond has been a subject of great concern [216]. However, by selectively breaking various carbon–carbon bonds in lignin and lignin monomers, specific compounds such as vanillin and syringaldehyde can be produced [217, 218].

#### Fractionation method for lignin extraction

The previous lignin extraction strategy was to fractionate lignin before catalytic depolymerization [219]. However, the limitation of the lignin solubility and the high temperature and/or acid/alkali adopted during the fractionation process would cause serious and irreversible condensation. A simple and avoidable condensation strategy has been developed, and it can retain the lignin structure via rapid flow-through fractionation. In the batch system, the lignin concentration is kept at a relatively low level by constantly adding fresh solvent to the system, and the dissolved lignin fragments are removed from the heating zone, which limits the extent of condensation and the structural changes in the lignin [76]. For instance, using *p*-toluenesulfonic acid (2.5 mol/L) in the flow-through reaction at 98 °C for 40 min, the yield of acid hydrolysate-dissolved lignin (AHL) reached 81.9%, with high β-O-4 bonds (80% retention) [220]. In addition, reductive catalytic fractionation (RCF), in which natural lignin from biomass was directly extracted by hydrogenolysis with a metal catalyst under a reductive atmosphere [221], was also developed to avoid condensation and prevent structural degradation. However, catalyst recovery and mass transfer in traditional batch reactors limit the application of RCF [76]. With technological innovation, a circulation system was invented to stabilize lignin intermediates and separate catalysts from biomass raw materials [201], which addressed the limitation of the RCF application in traditional batch reactors. RCF has two steps: the lignin in biomass is extracted using a polar-protic solvent, and then hydrogen donors and heterogeneous catalysts are used to cleave the C–O ether bond selectively [34]. Generally, under reductive conditions, the extracted lignin has more cleavable C–O bonds and fewer C–C bonds, resulting in a higher yield of aromatic monomers [222].

Additionally, many studies have explored oversimplified dimer model compounds that closely mimic the basic structure of real lignin, but they cannot reflect the complexity of side chains in natural lignin [79]. For example, β-O-4 model compounds lack the phenolic OH group, significantly influencing the reactivity of the experimental reagent to the model compound oxidation [79]. Therefore, the cleavage results under model compounds are difficult to achieve during the depolymerization of natural lignin, which still shows some significance for the further exploration of lignin extraction.

In summary, the recondensation of lignin is always the largest obstacle to the extraction of lignin and the production of aromatic monomers. Therefore, new findings can form stable intermediates or hinder the occurrence of recondensation or new reactors to improve reaction and separation efficiency, which must be further explored with rigor in the future.

### Genetic engineering technology

In recent decades, with the rise of synthetic biology, researchers have raised more interest in introducing genetic and metabolic engineering for the utilization of lignocellulosic biomass, which extends the scope and depth of the related research. New genetic manipulation technologies have been developed, providing the possibility of gene editing for a variety of organisms, which is no longer simply the overexpression of a single gene but the modification of parts of the structure, metabolic processes, and single enzymes of the selected organisms.

The cross-linking of lignin with cellulose and hemicellulose gives rise to plant structural rigidity to bear the weight of the entire plant, which is extremely important for the plant to maintain its own form. However, it is also the greatest obstacle to the use of lignocellulose feedstocks. In recent years, along with the invention of CRISPR technology and the subsequent report that CRISPR can be stably used for the genetic modification of some plants [223, 224], a strategy to reduce the difficulty of downstream use by lignin modification *in planta* was proposed. Lignin modification can achieve effects such as simplifying the structure of lignin and changing the content of the three structural units or total lignin without affecting the normal growth of plants, thereby reducing the difficulty of depolymerization, yielding more specific aromatic monomers, or increasing the calorific value of lignin (the ρ-hydroxyphenyl unit has the highest heating value), which enhances the potential application of lignin in high-value products and biofuels [225, 226]. Moreover, genetic engineering can also be used to improve polysaccharide properties and the composition of lignocellulose biomass, express enzymes that reduce the resistance of cell walls, improve stress tolerance and increase the yield stability of lignocellulose feedstocks [227, 228].

Enzymes are necessary for the biological pretreatment and saccharification processes, which have been studied extensively. Four enzymes reportedly can depolymerize lignin, namely laccases, manganese peroxidases (MnPs), lignin peroxidases (LiPs), and versatile peroxidases (VPs) [198], whose structures and efficiencies differ across species. In addition, natural microorganisms that can decompose lignin could also be applied for biological pretreatment, in which many bacterial species belonging to Actinomycetes, α-Proteobacteria and γ-Proteobacteria are included [229]. Furthermore, fungi are considered to be more effective at decomposing lignin [230], among which Basidiomycetes members are regarded as the only species that can completely degrade lignin [231]. Following pretreatment, the cellulase and xylanase required for the saccharification process also encounter the same problems as lignin-degrading enzymes, which can be solved by powerful technologies, including protein engineering and directed evolution, which can improve the properties of enzymes [232]; therefore, the enzymes with the best or the most stable activity could be selected for hydrolysis. Microorganisms often lack the high yields and productivity required for industrial applications [229]. Large amounts of stable and active enzymes can be obtained by genetic engineering, such as using a strong promoter or overexpression in host cells, which can not only reduce the cost of enzymes during the pretreatment and saccharification process but also pave the way for a one-step conversion strategy from lignocellulosic materials to high-value products [233–235].

Fermentation is a bioconversion process that converts sugars into high-value products and has been widely used to produce biofuels, one of the great products of lignocellulose biomass utilization research. Bioethanol can be produced by *Saccharomyces cerevisiae*. However, there has been a great deal of research on the mechanism by which yeast uses glucose to produce ethanol and on the improvement of ethanol production through genetic engineering based on its mechanisms in recent decades. Recently, *Saccharomyces cerevisiae* has been endowed with xylose utilization, as obtained through metabolic engineering, which further increases the production of bioethanol and chemicals [127, 236]. Similarly, this strategy has been adopted in the metabolic engineering of *Propionibacterium freudenreichii* subsp. *shermanii* for xylose fermentation [237]. Butanol-producing *Clostridium* can innately use glucose and xylose, similar to acetic acid, an inhibitor that is produced during some pretreatment methods, to produce biobutanol and chemicals by acetone–butanol–ethanol (ABE) fermentation. In the past decade, the application of genetic and metabolic engineering in butanol-producing *Clostridium* has been rapidly developed [99, 188, 189, 238–241] with the birth of ClosTron technology, which can be applied for *Clostridium* gene editing [242, 243], and the discovery of the butanol-producing metabolic mechanism of *Clostridium acetobutylicum* ATCC 824 [244]. Additionally, genetic and metabolic engineering has also been adopted in *Escherichia coli* for 2,3-butanediol production [245], *Neurospora crassa* for itaconic acid production [246], *Pseudomonas putida* KT2440 for substituted styrene bioproduct production [247], and other strains for biofuel and chemical production [98, 248–251]. Furthermore, some engineered microorganisms can also increase tolerance to inhibitors or upgrade lignin [7, 252].

As discussed above, the invention and improvement of efficient gene editing tools for different strains or lignocellulose feedstocks are the basis of genetic and metabolic engineering. Under normal circumstances, microorganisms that can naturally degrade lignin or cellulose cannot produce platform compounds, while microorganisms that can use sugars to produce high-value products cannot degrade lignin or cellulose. Therefore, to achieve one-step conversion from plant biomass to biofuels or chemicals, it is necessary to clone the required metabolic pathway-related genes into the corresponding strains through genetic engineering technology, during which the clarification of the synthetic and metabolic mechanism of the target compounds in microorganisms is critical in future research on the use of plant biomass. In addition, more in-depth research is needed to improve the resistance of plants and microorganisms and to reduce the lignin resistance of the plant cell wall.

## Conclusions

During the valorization of plant biomass into different types of high-value-added products, an increasing number of feedstocks have been adopted. The major development of the corresponding technologies is focused on changing the structure of lignocellulose to improve the efficiency of biomass conversion during downstream processes. Additionally, there are also great advances in lignin extraction treatments for producing aromatic-related value-added products. Nevertheless, it is necessary to alleviate or overcome the problems that occur during the valorization process, especially regarding cellulose utilization and aromatic monomer fractionation and improving genetic engineering technologies. In summary, the value-added products derived from plant biomass would grow in number and become more valuable along with the adoption of more plant biomass and the development of treatment technologies; meanwhile, greater social and environmental significance would achieve along with the valorization of plant biomass.

## Data Availability

Not applicable.

## References

[1] Wong WY, Lim S, Pang YL, Shuit SH, Chen WH, Lee KT (2020). Synthesis of renewable heterogeneous acid catalyst from oil palm empty fruit bunch for glycerol-free biodiesel production. Sci Total Environ..

[2] Bridgwater AV (2003). Renewable fuels and chemicals by thermal processing of biomass. Chem Eng J..

[3] Kumar V, Binod P, Sindhu R, Gnansounou E, Ahluwalia V (2018). Bioconversion of pentose sugars to value added chemicals and fuels: recent trends, challenges and possibilities. Bioresour Technol..

[4] Farzad S, Mandegari MA, Guo M, Haigh KF, Shah N, Görgens JF (2017). Multi-product biorefineries from lignocelluloses: a pathway to revitalisation of the sugar industry?. Biotechnol Biofuels..

[5] Rosales Calderon O, Arantes V (2019). A review on commercial-scale high-value products that can be produced alongside cellulosic ethanol. Biotechnol Biofuels..

[6] Bozell JJ, Petersen GR (2010). Technology development for the production of biobased products from biorefinery carbohydrates—the US Department of Energy’s “Top 10” revisited. Green Chem.

[7] Davis K, Moon TS (2020). Tailoring microbes to upgrade lignin. Curr Opin Chem Biol..

[8] Yu X, Wei Z, Lu Z, Pei H, Wang H (2019). Activation of lignin by selective oxidation: an emerging strategy for boosting lignin depolymerization to aromatics. Bioresour Technol..

[9] Mosier N, Wyman C, Dale B, Elander R, Lee YY, Holtzapple M, Ladisch M (2005). Features of promising technologies for pretreatment of lignocellulosic biomass. Bioresour Technol..

[10] Lin C-Y, Huang T-H (2012). Cost–benefit evaluation of using biodiesel as an alternative fuel for fishing boats in Taiwan. Mar Policy.

[11] Ge S, Wu Y, Peng W, Xia C, Mei C, Cai L, Shi SQ, Sonne C, Lam SS, Tsang YF (2020). High-pressure CO_2_ hydrothermal pretreatment of peanut shells for enzymatic hydrolysis conversion into glucose. Chem Eng J..

[12] Kargbo H, Harris JS, Phan AN (2021). “Drop-in” fuel production from biomass: Critical review on techno-economic feasibility and sustainability. Renew Sust Energ Rev..

[13] Mellor P, Lord RA, João E, Thomas R, Hursthouse A (2021). Identifying non-agricultural marginal lands as a route to sustainable bioenergy provision - a review and holistic definition. Renew Sust Energ Rev..

[14] Ussiri D, Lal R (2015). Miscanthus agronomy and bioenergy feedstock potential on minesoils. Biofuels.

[15] Jamsazzadeh Kermani Z, Shpigelman A, Pham HTT, Van Loey AM, Hendrickx ME (2015). Functional properties of citric acid extracted mango peel pectin as related to its chemical structure. Food Hydrocolloids.

[16] Ahmad MA, Afandi NS, Adegoke KA, Bello OS (2016). Optimization and batch studies on adsorption of malachite green dye using rambutan seed activated carbon. Desalin Water Treat..

[17] Queiroz LS, de Souza LKC, Thomaz KTC, Leite Lima ET, da Rocha Filho GN (2020). do Nascimento LAS, de Oliveira Pires LH, Faial KdCF, da Costa CEF: Activated carbon obtained from amazonian biomass tailings (acai seed): modification, characterization, and use for removal of metal ions from water. J Environ Manage..

[18] Dam JEG, Harmsen PFH: Coffee residues utilization**.** Wageningen UR - Food & Biobased Research; 2010.

[19] Aristizábal-Marulanda V, Solarte-Toro JC, Cardona Alzate CA: Study of biorefineries based on experimental data: production of bioethanol, biogas, syngas, and electricity using coffee-cut stems as raw material. Environ Sci Pollut Res. 2020. 10.1007/s11356-020-09804-y 32594433

[20] Wang S, Wang Z, Wang Y, Nie Q, Yi X, Ge W, Yang J, Xian M (2017). Production of isoprene, one of the high-density fuel precursors, from peanut hull using the high-efficient lignin-removal pretreatment method. Biotechnol Biofuels..

[21] Wang Z, Ning P, Hu L, Nie Q, Liu Y, Zhou Y, Yang J (2020). Efficient ethanol production from paper mulberry pretreated at high solid loading in Fed-nonisothermal-simultaneous saccharification and fermentation. Renew Energy..

[22] Waite JL (2017). Land reuse in support of renewable energy development. Land Use Policy.

[23] Lazar MD, Senila L, Dan M, Mihet M, Basile A, Iulianelli A, Dalena F, Veziroğlu TN (2019). Chapter 10 - Crude bioethanol reforming process: the advantage of a biosource exploitation. ethanol.

[24] Lam MK, Khoo CG, Lee KT, Pandey A, Chang J-S, Soccol CR, Lee D-J, Chisti Y (2019). Chapter 19 - Scale-up and commercialization of algal cultivation and biofuels production. Biofuels from Algae (Second Edition).

[25] Lin C-Y, Lu C (2021). Development perspectives of promising lignocellulose feedstocks for production of advanced generation biofuels: a review. Renew Sustain Energy Rev.

[26] Rodionova MV, Poudyal RS, Tiwari I, Voloshin RA, Zharmukhamedov SK, Nam HG, Zayadan BK, Bruce BD, Hou HJM, Allakhverdiev SI (2017). Biofuel production: Challenges and opportunities. Int J Hydrogen Energ..

[27] Budzinski M, Nitzsche R (2016). Comparative economic and environmental assessment of four beech wood based biorefinery concepts. Bioresour Technol..

[28] Hazwan Hussin M, Trache D, Chuin CTH, Nurul Fazita MR, Mohamad Haafiz MK, Hossain MS, Thomas S, Kumar Mishra R, Asiri AM (2019). Extraction of cellulose nanofibers and their eco-friendly polymer composites. Inamuddin.

[29] Liao JJ, Latif NHA, Trache D, Brosse N, Hussin MH (2020). Current advancement on the isolation, characterization and application of lignin. Int J Biol Macromol..

[30] Taherzadeh MJ, Karimi K (2008). Pretreatment of lignocellulosic wastes to improve ethanol and biogas production: a review. Int J Mol Sci..

[31] Zheng Y, Zhao J, Xu F, Li Y (2014). Pretreatment of lignocellulosic biomass for enhanced biogas production. Prog Energy Combust..

[32] Schutyser W, Renders T, Van den Bossche G, Van den Bosch S, Koelewijn S-F, Ennaert T, Sels BF: Catalysis in lignocellulosic biorefineries: The case of lignin conversion**.** In. Nanotechnology in Catalysis. 2017. p. 537-584.

[33] Bhatia SK, Jagtap SS, Bedekar AA, Bhatia RK, Patel AK, Pant D, Rajesh Banu J, Rao CV, Kim YG, Yang YH (2020). Recent developments in pretreatment technologies on lignocellulosic biomass: effect of key parameters, technological improvements, and challenges. Bioresour Technol..

[34] Korányi TI, Fridrich B, Pineda A, Barta K (2020). Development of 'Lignin-First' approaches for the valorization of lignocellulosic biomass. Molecules.

[35] Mottiar Y, Vanholme R, Boerjan W, Ralph J, Mansfield SD (2016). Designer lignins: harnessing the plasticity of lignification. Curr Opin Biotech..

[36] Chakar FS, Ragauskas AJ (2004). Review of current and future softwood kraft lignin process chemistry. Ind Crop Prod..

[37] Zakzeski J, Bruijnincx PCA, Jongerius AL, Weckhuysen BM (2010). The catalytic valorization of lignin for the production of renewable chemicals. Chem Rev..

[38] Cazacu G, Capraru M, Popa VI, Thomas S, Visakh PM, Mathew AP (2013). Advances concerning lignin utilization in new materials. Advances in Natural Polymers: Composites and Nanocomposites.

[39] Umezawa T (2018). Lignin modification *in planta* for valorization. Phytochem Rev..

[40] Mei Q, Shen X, Liu H, Han B (2019). Selectively transform lignin into value-added chemicals. Chin Chem Lett..

[41] Azman S, Khadem AF, van Lier JB, Zeeman G, Plugge CM (2015). Presence and role of anaerobic hydrolytic microbes in conversion of lignocellulosic Biomass for biogas production. Crit Rev Env Sci Tec..

[42] Lin W, Xing S, Jin Y, Lu X, Huang C, Yong Q (2020). Insight into understanding the performance of deep eutectic solvent pretreatment on improving enzymatic digestibility of bamboo residues. Bioresour Technol..

[43] de María P, Maugeri Z (2011). Ionic liquids in biotransformations: from proof-of-concept to emerging deep-eutectic-solvents. Curr Opin Chem Biol..

[44] Mbous YP, Hayyan M, Hayyan A, Wong WF, Hashim MA, Looi CY (2017). Applications of deep eutectic solvents in biotechnology and bioengineering—promises and challenges. Biotechnol Adv..

[45] Kumar N, Muley PD, Boldor D, Coty GG, Lynam JG (2019). Pretreatment of waste biomass in deep eutectic solvents: conductive heating versus microwave heating. Ind Crop Prod..

[46] Shen X-J, Wen J-L, Mei Q-Q, Chen X, Sun D, Yuan T-Q, Sun R-C (2019). Facile fractionation of lignocelluloses by biomass-derived deep eutectic solvent (DES) pretreatment for cellulose enzymatic hydrolysis and lignin valorization. Green Chem.

[47] Wang Z-K, Li H, Lin X-C, Tang L, Chen J-J, Mo J-W, Yu R-S, Shen X-J (2020). Novel recyclable deep eutectic solvent boost biomass pretreatment for enzymatic hydrolysis. Bioresour Technol..

[48] Huang C, Zhan Y, Cheng J, Wang J, Meng X, Zhou X, Fang G, Ragauskas AJ (2021). Facilitating enzymatic hydrolysis with a novel guaiacol-based deep eutectic solvent pretreatment. Bioresour Technol..

[49] Zhou X, Huang T, Liu J, Gao H, Bian H, Wang R, Huang C, Sha J, Dai H (2021). Recyclable deep eutectic solvent coupling sodium hydroxide post-treatment for boosting woody/herbaceous biomass conversion at mild condition. Bioresour Technol..

[50] Xu H, Kong Y, Peng J, Song X, Liu Y, Su Z, Li B, Gao C, Tian W (2021). Comprehensive analysis of important parameters of choline chloride-based deep eutectic solvent pretreatment of lignocellulosic biomass. Bioresour Technol..

[51] Morais ES, Da Costa Lopes AM, Freire MG, Freire CSR, Silvestre AJD (2021). Unveiling modifications of biomass polysaccharides during thermal treatment in cholinium chloride: Lactic acid deep eutectic solvent. Chemsuschem.

[52] Ma Q, Gao X, Bi X, Xia M, Han Q, Peng M, Tu L, Yang Y, Shen Y, Wang M (2021). Combination of steam explosion and ionic liquid pretreatments for efficient utilization of fungal chitin from citric acid fermentation residue. Biomass Bioenerg..

[53] Zheng Y, Pan Z, Zhang R (2008). Overview of biomass pretreatment for cellulosic ethanol. Int J Agr Biol Eng..

[54] Paralikar K, Betrabet S (1977). Electron-diffraction technique for determination of cellulose crystallinity. J Appl Polym Sci..

[55] Tomás-Pejó E, Alvira P, Ballesteros M, Negro MJ, Pandey A, Larroche C, Ricke SC, Dussap C-G, Gnansounou E (2011). Chapter 7 - Pretreatment technologies for lignocellulose-to-bioethanol conversion. Biofuels.

[56] Smichi N, Messaoudi Y, Allaf K, Gargouri M (2020). Steam explosion (SE) and instant controlled pressure drop (DIC) as thermo-hydro-mechanical pretreatment methods for bioethanol production. Bioprocess Biosyst Eng..

[57] Xia M, Peng M, Xue D, Cheng Y, Li C, Wang D, Lu K, Zheng Y, Xia T, Song J, Wang M (2020). Development of optimal steam explosion pretreatment and highly effective cell factory for bioconversion of grain vinegar residue to butanol. Biotechnol Biofuels..

[58] Bhatia R, Winters A, Bryant DN, Bosch M, Clifton-Brown J, Leak D, Gallagher J (2020). Pilot-scale production of xylo-oligosaccharides and fermentable sugars from *Miscanthus* using steam explosion pretreatment. Bioresour Technol..

[59] Zeng ZK, Jang JC, Shurson GC, Thakral S, Urriola PE (2021). Ammonia fiber expansion increases *in vitro* digestibility and fermentability of corn distillers dried grains with solubles with or without carbohydrases. Anim Feed Sci Tech..

[60] Zhao C, Shao Q, Chundawat SPS (2020). Recent advances on ammonia-based pretreatments of lignocellulosic biomass. Bioresour Technol..

[61] Cai C, Wang L, Wang G, Hao J, Bai X, Wang Z, Wang D (2020). Effects of dry explosion pretreatment on physicochemical and fuel properties of hybrid pennisetum (*Pennisetum americanum* × *n*). Bioresour Technol..

[62] Zhu JY, Pan XJ, Wang GS, Gleisner R (2009). Sulfite pretreatment (SPORL) for robust enzymatic saccharification of spruce and red pine. Bioresour Technol..

[63] Zhu J, Pan XJ (2010). Woody biomass pretreatment for cellulosic ethanol production: technology and energy consumption evaluation. Bioresour Technol..

[64] Zhu JY, Chandra MS, Gu F, Gleisner R, Reiner R, Sessions J, Marrs G, Gao J, Anderson D (2015). Using sulfite chemistry for robust bioconversion of douglas-fir forest residue to bioethanol at high titer and lignosulfonate: a pilot-scale evaluation. Bioresour Technol..

[65] vom Stein T, Grande PM, Kayser H, Sibilla F, Leitner W (2011). Domínguez de María P: From biomass to feedstock: one-step fractionation of lignocellulose components by the selective organic acid-catalyzed depolymerization of hemicellulose in a biphasic system. Green Chem.

[66] Weidener D, Dama M, Dietrich SK, Ohrem B, Pauly M, Leitner W (2020). Multiscale analysis of lignocellulose recalcitrance towards OrganoCat pretreatment and fractionation. Biotechnol Biofuels..

[67] Weidener D, Klose H, Leitner W, Schurr U, Usadel B (2018). One-Step lignocellulose fractionation by using 2,5-furandicarboxylic acid as a biogenic and recyclable catalyst. Chemsuschem.

[68] Morone A, Pandey RA, Chakrabarti T (2017). Evaluation of OrganoCat process as a pretreatment during bioconversion of rice straw. Ind Crop Prod..

[69] Grande PM, Viell J, Theyssen N, Marquardt W (2015). Fractionation of lignocellulosic biomass using the OrganoCat process. Green Chem.

[70] Kalogiannis KG, Karnaouri A, Michailof C, Tzika AM, Asimakopoulou G, Topakas E, Lappas AA (2020). OxiOrganosolv: a novel acid free oxidative organosolv fractionation for lignocellulose fine sugar streams. Bioresour Technol..

[71] Katsimpouras C, Kalogiannis KG, Kalogianni A, Lappas AA, Topakas E (2017). Production of high concentrated cellulosic ethanol by acetone/water oxidized pretreated beech wood. Biotechnol Biofuels..

[72] Liu C, Liu X, He Y, An X, Fan D, Wu Z (2021). Microwave-assisted catalytic pyrolysis of apple wood to produce biochar: Co-pyrolysis behavior, pyrolysis kinetics analysis and evaluation of microbial carriers. Bioresour Technol..

[73] Flores EMM, Cravotto G, Bizzi CA, Santos D, Iop GD (2021). Ultrasound-assisted biomass valorization to industrial interesting products: state-of-the-art, perspectives and challenges. Ultrasonics Sonochem.

[74] Pereira Marques F, Lima Soares AK, Lomonaco D, Alexandre e Silva LM, Tédde Santaella S, de Freitas Rosa M, Carrhá-Leitão R (2021). Steam explosion pretreatment improves acetic acid organosolv delignification of oil palm mesocarp fibers and sugarcane bagasse. Int J Biol Macromol..

[75] Bhatia R, Lad JB, Bosch M, Bryant DN, Leak D, Hallett JP, Franco TT, Gallagher JA (2021). Production of oligosaccharides and biofuels from *Miscanthus* using combinatorial steam explosion and ionic liquid pretreatment. Bioresour Technol..

[76] Xu J, Li C, Dai L, Xu C, Zhong Y, Yu F, Si C (2020). Biomass fractionation and lignin fractionation towards lignin valorization. Chemsuschem.

[77] Mai C, Majcherczyk A, Hüttermann A (2000). Chemo-enzymatic synthesis and characterization of graft copolymers from lignin and acrylic compounds. Enzyme Microb Tech..

[78] Luo H, Abu-Omar MM, Abraham MA (2017). Chemicals from lignin. Encyclopedia of sustainable technologies.

[79] Vangeel T, Schutyser W, Renders T, Sels BF (2018). Perspective on lignin oxidation: advances, challenges, and future directions. Top Curr Chem (Cham)..

[80] Kang Y, Lu X, Zhang G, Yao X, Xin J, Yang S, Yang Y, Xu J, Feng M, Zhang S (2019). Metal-free photochemical degradation of lignin-derived aryl ethers and lignin by autologous radicals through ionic liquid induction. Chemsuschem.

[81] Han G, Yan T, Zhang W, Zhang YC, Lee DY, Cao Z, Sun Y (2019). Highly selective photocatalytic valorization of lignin model compounds using ultrathin metal/CdS. ACS Catal.

[82] Lin J, Wu X, Xie S, Chen L, Zhang Q, Deng W, Wang Y (2019). Visible-light-driven cleavage of C−O linkage for lignin valorization to functionalized aromatics. Chemsuschem.

[83] Cai P, Fan H, Cao S, Qi J, Zhang S, Li G (2018). Electrochemical conversion of corn stover lignin to biomass-based chemicals between Cu/NiMoCo cathode and Pb/PbO2 anode in alkali solution. Electrochim Acta.

[84] Xiang Z, Han W, Deng J, Zhu W, Zhang Y, Wang H (2020). Photocatalytic conversion of lignin into chemicals and fuels. Chemsuschem.

[85] Luo N, Wang M, Li H, Zhang J, Liu H, Wang F (2016). Photocatalytic oxidation–hydrogenolysis of lignin β-O-4 models via a dual light wavelength switching strategy. ACS Catal.

[86] Rinaldi R, Jastrzebski R, Clough MT, Ralph J, Kennema M, Bruijnincx PC, Weckhuysen BM (2016). Paving the way for lignin valorisation: recent advances in bioengineering, biorefining and catalysis. Angew Chem Int Edit..

[87] Nguyen TY, Cai CM, Kumar R, Wyman CE (2015). Co-solvent pretreatment reduces costly enzyme requirements for high sugar and ethanol yields from lignocellulosic biomass. Chemsuschem.

[88] Nguyen TY, Cai CM, Kumar R, Wyman CE (2017). Overcoming factors limiting high-solids fermentation of lignocellulosic biomass to ethanol. P Natl Acad Sci USA.

[89] Matouš J, Novák J, Šobr J, Pick J (1972). Phase equilibriums in the system tetrahydrofuran(1)–water(2). Collect Czech Chem C..

[90] Li J, Zhang W, Xu S, Hu C (2020). The roles of H_2_O/tetrahydrofuran system in lignocellulose valorization. Front Chem..

[91] Zhuo S, Peng B, Yan X, Zhang K, Si M, Liu M, Shi Y (2018). Conquering lignin recalcitrance by *Pandoraea* sp. B-6 to improve co-solvent pretreatment of corn stover. Process Biochem..

[92] Petridis L, Smith JC (2018). Molecular-level driving forces in lignocellulosic biomass deconstruction for bioenergy. Nat Rev Chem..

[93] Zhang H, Liu X, Li J, Jiang Z, Hu C (2018). Performances of several solvents on the cleavage of inter- and intramolecular linkages of lignin in corncob residue. Chemsuschem.

[94] Cai CM, Zhang T, Kumar R, Wyman CE (2013). THF co-solvent enhances hydrocarbon fuel precursor yields from lignocellulosic biomass. Green Chem.

[95] Damm T, Grande PM, Jablonowski ND, Thiele B, Disko U, Mann U, Schurr U, Leitner W, Usadel B (2017). Domínguez de María P, Klose H: OrganoCat pretreatment of perennial plants: Synergies between a biogenic fractionation and valuable feedstocks. Bioresour Technol..

[96] Holtz A, Weidener D, Leitner W, Klose H, Grande PM, Jupke A (2020). Process development for separation of lignin from OrganoCat lignocellulose fractionation using antisolvent precipitation. Sep Purif Technol..

[97] Wen J-L, Sun S-L, Yuan T-Q, Sun R-C (2015). Structural elucidation of whole lignin from *Eucalyptus* based on preswelling and enzymatic hydrolysis. Green Chem.

[98] Francois JM, Alkim C, Morin N (2020). Engineering microbial pathways for production of bio-based chemicals from lignocellulosic sugars: current status and perspectives. Biotechnol Biofuels..

[99] Zhang L, Zhao R, Jia D, Jiang W, Gu Y (2020). Engineering *Clostridium ljungdahlii* as the gas-fermenting cell factory for the production of biofuels and biochemicals. Curr Opin Chem Biol..

[100] Curvello R, Raghuwanshi VS, Garnier G (2019). Engineering nanocellulose hydrogels for biomedical applications. Adv Colloid Interface Sci..

[101] Kwon G, Bhatnagar A, Wang H, Kwon EE, Song H (2020). A review of recent advancements in utilization of biomass and industrial wastes into engineered biochar. J Hazard Mater..

[102] Guan W, Tsang CW, Lin CSK, Len C, Hu H, Liang C (2020). A review on high catalytic efficiency of solid acid catalysts for lignin valorization. Bioresour Technol..

[103] Xin H, Hu X, Cai C, Wang H, Zhu C, Li S, Xiu Z, Zhang X, Liu Q, Ma L (2020). Catalytic production of oxygenated and hydrocarbon chemicals from cellulose hydrogenolysis in aqueous phase. Front Chem..

[104] Yuan D, Li L, Li F, Wang Y, Wang F, Zhao N, Xiao F (2019). Solvent-free production of isosorbide from sorbitol catalyzed by a polymeric solid acid. Chemsuschem.

[105] Huber GW, Shabaker JW, Dumesic JA (2003). Raney Ni-Sn catalyst for H_2_ production from biomass-derived hydrocarbons. Science.

[106] Davda RR, Dumesic JA (2004). Renewable hydrogen by aqueous-phase reforming of glucose. Chem Commun..

[107] Park S-A, Im C, Oh DX, Hwang SY, Jegal J, Kim JH, Chang Y-W, Jeon H, Park J (2019). Study on the synthetic characteristics of biomass-derived isosorbide-based poly(arylene ether ketone)s for sustainable super engineering plastic. Molecules.

[108] Phan D-N, Lee H, Choi D, Kang C-Y, Im SS, Kim IS (2018). Fabrication of two polyester nanofiber types containing the biobased monomer isosorbide: poly (ethylene glycol 1,4-cyclohexane dimethylene isosorbide terephthalate) and poly (1,4-cyclohexane dimethylene isosorbide terephthalate). Nanomaterials.

[109] Li N, Huber GW (2010). Aqueous-phase hydrodeoxygenation of sorbitol with Pt/SiO2–Al2O3: identification of reaction intermediates. J Catal..

[110] Yang Y, Zhang W, Yang F, Zhou B, Zeng D, Zhang N, Zhao G, Hao S, Zhang X (2018). Ru nanoparticles dispersed on magnetic yolk-shell nanoarchitectures with FeO core and sulfoacid-containing periodic mesoporous organosilica shell as bifunctional catalysts for direct conversion of cellulose to isosorbide. Nanoscale.

[111] Bonnin I, Mereau R, Tassaing T, De Oliveira VK (2020). One-pot synthesis of isosorbide from cellulose or lignocellulosic biomass: a challenge?. Beilstein J Org Chem..

[112] Gu M, Shen Z, Yang L, Dong W, Kong L, Zhang W, Peng B-Y, Zhang Y (2019). Reaction route selection for cellulose hydrogenolysis into C_2_/C_3_ Glycols by ZnO-modified Ni-W/β-zeolite catalysts. Sci Rep.

[113] Ryu YW, Park CY, Park JB, Kim SY, Seo JH (2000). Optimization of erythritol production by *Candida magnoliae* in fed-batch culture. J Ind Microbiol Biot..

[114] Nakagawa Y, Kasumi T, Ogihara J, Tamura M, Arai T, Tomishige K (2020). Erythritol: another C_4_ platform chemical in biomass refinery. ACS Omega.

[115] Bu CY, Yan YX, Zou LH, Zheng ZJ, Ouyang J (2020). One-pot biosynthesis of furfuryl alcohol and lactic acid via a glucose coupled biphasic system using single *Bacillus coagulans* NL01. Bioresour Technol..

[116] Murali N, Fernandez S, Ahring BK (2017). Fermentation of wet-exploded corn stover for the production of volatile fatty acids. Bioresour Technol..

[117] Baumann I, Westermann P (2016). Microbial production of short chain fatty acids from lignocellulosic biomass: current processes and market. Biomed Res Int..

[118] Alsafadi D, Ibrahim MI, Alamry KA, Hussein MA, Mansour A (2020). Utilizing the crop waste of date palm fruit to biosynthesize polyhydroxyalkanoate bioplastics with favorable properties. Sci Total Environ..

[119] Choi SY, Rhie MN, Kim HT, Joo JC, Cho IJ, Son J, Jo SY, Sohn YJ, Baritugo K-A, Pyo J (2020). Metabolic engineering for the synthesis of polyesters: a 100-year journey from polyhydroxyalkanoates to non-natural microbial polyesters. Metab Eng..

[120] Sirohi R, Prakash Pandey J, Kumar Gaur V, Gnansounou E, Sindhu R (2020). Critical overview of biomass feedstocks as sustainable substrates for the production of polyhydroxybutyrate (PHB). Bioresour Technol..

[121] Xu C, Paone E, Rodríguez-Padrón D, Luque R, Mauriello F (2020). Recent catalytic routes for the preparation and the upgrading of biomass derived furfural and 5-hydroxymethylfurfural. Chem Soc Rev..

[122] Kisszekelyi P, Hardian R, Vovusha H, Chen B, Zeng X, Schwingenschlögl U, Kupai J, Szekely G (2020). Selective electrocatalytic oxidation of biomass-derived 5-hydroxymethylfurfural to 2,5-diformylfuran: from mechanistic investigations to catalyst recovery. Chemsuschem.

[123] Körner S, Albert J, Held C (2019). Catalytic low-temperature dehydration of fructose to 5-hydroxymethylfurfural using acidic deep eutectic solvents and polyoxometalate catalysts. Front Chem..

[124] Park H, Jeong D, Shin M, Kwak S, Oh EJ, Ko JK, Kim SR (2020). Xylose utilization in *Saccharomyces cerevisiae* during conversion of hydrothermally pretreated lignocellulosic biomass to ethanol. Appl Microbiol Biot..

[125] Sundar MSL, Susmitha A, Rajan D, Hannibal S, Sasikumar K, Wendisch VF, Nampoothiri KM (2020). Heterologous expression of genes for bioconversion of xylose to xylonic acid in *Corynebacterium glutamicum* and optimization of the bioprocess. AMB Expr.

[126] Zhang Y, Lane S, Chen J-M, Hammer SK, Luttinger J, Yang L, Jin Y-S, Avalos JL (2019). Xylose utilization stimulates mitochondrial production of isobutanol and 2-methyl-1-butanol in *Saccharomyces cerevisiae*. Biotechnol Biofuels..

[127] Kwak S, Jo JH, Yun EJ, Jin Y-S, Seo J-H (2019). Production of biofuels and chemicals from xylose using native and engineered yeast strains. Biotechnol Adv..

[128] Felipe Hernández-Pérez A, de Arruda PV, Sene L, da Silva SS, Kumar Chandel A (2019). de Almeida Felipe MdG: Xylitol bioproduction: state-of-the-art, industrial paradigm shift, and opportunities for integrated biorefineries. Crit Rev Biotechnol..

[129] Xu Y, Chi P, Bilal M, Cheng H (2019). Biosynthetic strategies to produce xylitol: an economical venture. Appl Microbiol Biot..

[130] Tippkötter N, Duwe AM, Wiesen S, Sieker T, Ulber R (2014). Enzymatic hydrolysis of beech wood lignocellulose at high solid contents and its utilization as substrate for the production of biobutanol and dicarboxylic acids. Bioresour Technol..

[131] Liu H, Hu H, Jin Y, Yue X, Deng L, Wang F, Tan T (2017). Co-fermentation of a mixture of glucose and xylose to fumaric acid by *Rhizopus arrhizus RH 7–13-9#*. Bioresour Technol..

[132] Kumar G, Shobana S, Chen W-H, Bach Q-V, Kim S-H, Atabani AE, Chang J-S (2017). A review of thermochemical conversion of microalgal biomass for biofuels: chemistry and processes. Green Chem.

[133] Kapoor R, Ghosh P, Kumar M, Sengupta S, Gupta A, Kumar SS, Vijay V, Kumar V, Kumar Vijay V, Pant D (2020). Valorization of agricultural waste for biogas based circular economy in India: a research outlook. Bioresour Technol..

[134] Vivek N, Sindhu R, Madhavan A, Anju AJ, Castro E, Faraco V, Pandey A, Binod P (2017). Recent advances in the production of value added chemicals and lipids utilizing biodiesel industry generated crude glycerol as a substrate – Metabolic aspects, challenges and possibilities: an overview. Bioresour Technol..

[135] André A, Diamantopoulou P, Philippoussis A, Sarris D, Komaitis M, Papanikolaou S (2010). Biotechnological conversions of bio-diesel derived waste glycerol into added-value compounds by higher fungi: production of biomass, single cell oil and oxalic acid. Ind Crop Prod..

[136] Garlapati VK, Shankar U, Budhiraja A (2016). Bioconversion technologies of crude glycerol to value added industrial products. Biotechnol Rep.

[137] Morgunov IG, Kamzolova SV, Lunina JN (2013). The citric acid production from raw glycerol by *Yarrowia lipolytic*a yeast and its regulation. Appl Microbiol Biotechnol.

[138] Hu S, Li Y (2014). Two-step sequential liquefaction of lignocellulosic biomass by crude glycerol for the production of polyols and polyurethane foams. Bioresour Technol..

[139] André A, Chatzifragkou A, Diamantopoulou P, Sarris D, Philippoussis A, Galiotou-Panayotou M, Komaitis M, Papanikolaou S (2009). Biotechnological conversions of bio-diesel-derived crude glycerol by *Yarrowia lipolytica* strains. Eng Life Sci..

[140] Yuan X, Tu S, Lin J, Yang L, Shen H, Wu M (2020). Combination of the CRP mutation and ptsG deletion in Escherichia coli to efficiently synthesize xylitol from corncob hydrolysates. Appl Microbiol Biotechnol.

[141] Liu X, Yu X, Xia J, Lv J, Xu J, Dai B, Xu X, Xu J (2017). Erythritol production by *Yarrowia lipolytica* from okara pretreated with the in-house enzyme pools of fungi. Bioresour Technol..

[142] Papanikolaou S (2008). 1,3-Propanediol and citric acid production from glycerol-containing waste discharged after bio-diesel manufacturing process. Curr Top Bioprocess Food Indus..

[143] Wu Y, Jiang L, Lin Y, Qian L, Xu F, Lang X, Fan S, Zhao Z, Li H (2019). Novel crude glycerol pretreatment for selective saccharification of sugarcane bagasse via fast pyrolysis. Bioresour Technol..

[144] Jiang L, Zheng A, Zhao Z, He F, Li H (2015). Comprehensive utilization of glycerol from sugarcane bagasse pretreatment to fermentation. Bioresour Technol..

[145] Intasit R, Cheirsilp B, Louhasakul Y, Boonsawang P (2020). Consolidated bioprocesses for efficient bioconversion of palm biomass wastes into biodiesel feedstocks by oleaginous fungi and yeasts. Bioresour Technol..

[146] Haq I, Mazumder P, Kalamdhad AS (2020). Recent advances in removal of lignin from paper industry wastewater and its industrial applications - a review. Bioresour Technol..

[147] Bridgwater AV (2012). Review of fast pyrolysis of biomass and product upgrading. Biomass Bioenerg..

[148] Dickerson T, Soria J (2013). Catalytic fast pyrolysis: a review.. Energies.

[149] Mohan D, Pittman CU, Steele PH (2006). Pyrolysis of wood/biomass for bio-oil: a critical review. Energy Fuel..

[150] Tshikesho RS, Kumar A, Huhnke RL, Apblett A (2019). Catalytic co-pyrolysis of red cedar with methane to produce upgraded bio-oil. Bioresour Technol..

[151] Quispe I, Navia R, Kahhat R (2017). Energy potential from rice husk through direct combustion and fast pyrolysis: a review. Waste Manag..

[152] Zhang S, Yang X, Zhang H, Chu C, Zheng K, Ju M, Liu L (2019). Liquefaction of biomass and upgrading of bio-oil: a review. Molecules.

[153] Kim J-S (2015). Production, separation and applications of phenolic-rich bio-oil - a review. Bioresour Technol..

[154] Li Y, Xing B, Ding Y, Han X, Wang S (2020). A critical review of the production and advanced utilization of biochar via selective pyrolysis of lignocellulosic biomass. Bioresour Technol..

[155] Ponnusamy VK, Nagappan S, Bhosale RR, Lay CH, Duc Nguyen D, Pugazhendhi A, Chang SW, Kumar G (2020). Review on sustainable production of biochar through hydrothermal liquefaction: physico-chemical properties and applications. Bioresour Technol..

[156] Wang D, Jiang P, Zhang H, Yuan W (2020). Biochar production and applications in agro and forestry systems: a review. Sci Total Environ..

[157] Zoroufchi Benis K, Motalebi Damuchali A, Soltan J, McPhedran KN (2020). Treatment of aqueous arsenic - a review of biochar modification methods. Sci Total Environ..

[158] Wang J, Wang S (2019). Preparation, modification and environmental application of biochar: a review. J Clean Prod..

[159] Tam NTM, Liu YG, Bashir H, Zhang P, Liu SB, Tan X, Dai MY, Li MF (2020). Synthesis of porous biochar containing graphitic carbon derived from lignin content of forestry biomass and its application for the removal of diclofenac sodium from aqueous solution. Front Chem..

[160] Li J, Yu G, Pan L, Li C, You F, Wang Y (2020). Ciprofloxacin adsorption by biochar derived from co-pyrolysis of sewage sludge and bamboo waste. Environ Sci Pollut Res..

[161] Singh S, Kumar V, Datta S, Dhanjal DS, Sharma K, Samuel J, Singh J (2020). Current advancement and future prospect of biosorbents for bioremediation. Sci Total Environ..

[162] Yek PNY, Peng W, Wong CC, Liew RK, Ho YL, Wan Mahari WA, Azwar E, Yuan TQ, Tabatabaei M, Aghbashlo M (2020). Engineered biochar via microwave CO_2_ and steam pyrolysis to treat carcinogenic Congo red dye. J Hazard Mater..

[163] Cheng S, Chen T, Xu W, Huang J, Jiang S, Yan B (2020). Application research of biochar for the remediation of soil heavy metals contamination: a review. Molecules.

[164] Kim JY, Oh S, Park YK (2020). Overview of biochar production from preservative-treated wood with detailed analysis of biochar characteristics, heavy metals behaviors, and their ecotoxicity. J Hazard Mater..

[165] Kumar A, Saini K, Bhaskar T (2020). Advances in design strategies for preparation of biochar based catalytic system for production of high value chemicals. Bioresour Technol..

[166] Guo F, Jia X, Liang S, Zhou N, Chen P, Ruan R (2020). Development of biochar-based nanocatalysts for tar cracking/reforming during biomass pyrolysis and gasification. Bioresour Technol..

[167] Sun X, Atiyeh HK, Li M, Chen Y (2020). Biochar facilitated bioprocessing and biorefinery for productions of biofuel and chemicals: a review. Bioresour Technol..

[168] Liu X, Wang Z, Yan Y, Yu X, Zhao P, Wang X, Hu L, Xu J, Xu J (2020). Novel strategy of incorporating biochar in solid-state fermentation for enhancing erythritol production by forming "microzones". Bioresour Technol..

[169] Amoah-Antwi C, Kwiatkowska-Malina J, Thornton SF, Fenton O, Malina G, Szara E (2020). Restoration of soil quality using biochar and brown coal waste: a review. Sci Total Environ..

[170] Ryms M, Januszewicz K, Kazimierski P, Łuczak J, Klugmann-Radziemska E, Lewandowski WM (2020). Post-pyrolytic carbon as a phase change materials (PCMs) carrier for application in building materials. Materials..

[171] Ding Y, Greiner M, Schlögl R, Heumann S (2020). A metal-free electrode: from biomass-derived carbon to hydrogen. Chemsuschem.

[172] Shrestha RG, Maji S, Shrestha LK, Ariga K (2020). Nanoarchitectonics of nanoporous carbon materials in supercapacitors applications. Nanomaterials.

[173] Buaki-Sogó M, Zubizarreta L, García-Pellicer M, Quijano-López A (2020). Sustainable carbon as efficient support for metal-based nanocatalyst: applications in energy harvesting and storage. Molecules.

[174] Han J, Kim H (2008). The reduction and control technology of tar during biomass gasification/pyrolysis: an overview. Renew Sustain Energy Rev..

[175] Brown RC: Introduction to thermochemical processing of biomass into fuels, chemicals, and power**.** In: Stevens CV, editors. Thermochemical Processing of Biomass. 2011. p. 1-12.

[176] Dos Santos FJ, de Oliveira D, Maldonado RR, Kamimura ES, Furigo A (2020). Enzymatic pretreatment and anaerobic co-digestion as a new technology to high-methane production. Appl Microbiol Biot..

[177] Rena MB (2020). Zacharia K, Yadav S, Machhirake NP, Kim SH, Lee BD, Jeong H, Singh L, Kumar S, Kumar R: Bio-hydrogen and bio-methane potential analysis for production of bio-hythane using various agricultural residues. Bioresour Technol..

[178] Valenti F, Porto SMC, Selvaggi R, Pecorino B (2018). Evaluation of biomethane potential from by-products and agricultural residues co-digestion in southern Italy. J Environ Manage..

[179] Arreola-Vargas J, Flores-Larios A, González-Álvarez V, Corona-González RI, Méndez-Acosta HO (2016). Single and two-stage anaerobic digestion for hydrogen and methane production from acid and enzymatic hydrolysates of *Agave* tequilana bagasse. Int J Hydrogen Energy.

[180] Liew FM, Köpke M, Simpson SD: Gas fermentation for commercial biofuels production**.** 2013.

[181] Sharma B, Larroche C, Dussap CG (2020). Comprehensive assessment of 2G bioethanol production. Bioresour Technol..

[182] Liguori R, Faraco V (2016). Biological processes for advancing lignocellulosic waste biorefinery by advocating circular economy. Bioresour Technol..

[183] Zhang Z, Song J, Han B (2017). Catalytic transformation of lignocellulose into chemicals and fuel products in ionic liquids. Chem Rev..

[184] Pfromm PH, Amanor-Boadu V, Nelson R, Vadlani P, Madl R (2010). Bio-butanol vs bio-ethanol: a technical and economic assessment for corn and switchgrass fermented by yeast or *Clostridium acetobutylicum*. Biomass Bioenergy..

[185] Wen Z, Ledesma-Amaro R, Lin J, Jiang Y, Yang S (2019). Improved -butanol production from *Clostridium cellulovorans* by integrated metabolic and evolutionary engineering. Appl Environ Microb..

[186] Luo H, Zheng P, Bilal M, Xie F, Zeng Q, Zhu C, Yang R, Wang Z (2020). Efficient bio-butanol production from lignocellulosic waste by elucidating the mechanisms of *Clostridium acetobutylicum* response to phenolic inhibitors. Sci Total Environ..

[187] Zhao T, Yasuda K, Tashiro Y, Darmayanti RF, Sakai K, Sonomoto K (2019). Semi-hydrolysate of paper pulp without pretreatment enables a consolidated fermentation system with in situ product recovery for the production of butanol. Bioresour Technol..

[188] Li S, Huang L, Ke C, Pang Z, Liu L (2020). Pathway dissection, regulation, engineering and application: lessons learned from biobutanol production by solventogenic clostridia. Biotechnol Biofuels..

[189] Zhao T, Tashiro Y, Sonomoto K (2019). Smart fermentation engineering for butanol production: designed biomass and consolidated bioprocessing systems. Appl Microbiol Biot..

[190] Vermaas JV, Petridis L, Qi X, Schulz R, Lindner B, Smith JC (2015). Mechanism of lignin inhibition of enzymatic biomass deconstruction. Biotechnol Biofuels..

[191] Lan W, Luterbacher JS (2019). Preventing lignin condensation to facilitate aromatic monomer production. Chimia.

[192] Kim KH, Kim CS (2018). Recent efforts to prevent undesirable reactions from fractionation to depolymerization of lignin: toward maximizing the value from lignin. Front Energy Res..

[193] Millati R, Wikandari R, Ariyanto T, Putri RU, Taherzadeh MJ (2020). Pretreatment technologies for anaerobic digestion of lignocelluloses and toxic feedstocks. Bioresour Technol..

[194] Carrere H, Antonopoulou G, Affes R, Passos F, Battimelli A, Lyberatos G, Ferrer I (2016). Review of feedstock pretreatment strategies for improved anaerobic digestion: from lab-scale research to full-scale application. Bioresour Technol..

[195] Vieira S, Barros MV, Sydney ACN, Piekarski CM, de Francisco AC, Vandenberghe LPS, Sydney EB (2020). Sustainability of sugarcane lignocellulosic biomass pretreatment for the production of bioethanol. Bioresour Technol..

[196] Islam MK, Wang H, Rehman S, Dong C, Hsu HY, Lin CSK, Leu SY (2020). Sustainability metrics of pretreatment processes in a waste derived lignocellulosic biomass biorefinery. Bioresour Technol..

[197] Bugg TDH, Williamson JJ, Rashid GMM (2020). Bacterial enzymes for lignin depolymerisation: new biocatalysts for generation of renewable chemicals from biomass. Curr Opin Chem Biol..

[198] Pollegioni L, Tonin F, Rosini E (2015). Lignin-degrading enzymes. Febs J..

[199] Zabed H, Akter S, Yun J, Zhang G, Awad F, Qi X, Sahu J (2019). Recent advances in biological pretreatment of microalgae and lignocellulosic biomass for biofuel production. Renew Sustain Energy Rev..

[200] Li Q, Serem WK, Dai W, Yue Y, Naik MT, Xie S, Karki P, Liu L, Sue H-J, Liang H (2017). Molecular weight and uniformity define the mechanical performance of lignin-based carbon fiber. J Mater Chem A..

[201] Liu X, Bouxin FP, Fan J, Budarin VL, Hu C, Clark JH (2020). Recent advances in the catalytic depolymerization of lignin towards phenolic chemicals: a review. Chemsuschem.

[202] Lahive CW, Deuss PJ, Lancefield CS, Sun Z, Cordes DB, Young CM, Tran F, Slawin AMZ, de Vries JG, Kamer PCJ (2016). Advanced model compounds for understanding acid-catalyzed lignin depolymerization: identification of renewable aromatics and a lignin-derived solvent. J Am Chem Soc..

[203] Sturgeon MR, Kim S, Lawrence K, Paton RS, Chmely SC, Nimlos M, Foust TD, Beckham GT (2014). A mechanistic investigation of acid-catalyzed cleavage of aryl-ether linkages: implications for lignin depolymerization in acidic environments. ACS Sustain Chem Eng..

[204] Zhang C, Li H, Lu J, Zhang X, MacArthur KE, Heggen M, Wang F (2017). Promoting lignin depolymerization and restraining the condensation via an oxidation−hydrogenation strategy. ACS Catal.

[205] Rahimi A, Ulbrich A, Coon JJ, Stahl SS (2014). Formic-acid-induced depolymerization of oxidized lignin to aromatics. Nature.

[206] Rahimi A, Azarpira A, Kim H, Ralph J, Stahl SS (2013). Chemoselective metal-free aerobic alcohol oxidation in lignin. J Am Chem Soc..

[207] Liu H, Li H, Lu J, Zeng S, Wang M, Luo N, Xu S, Wang F (2018). Photocatalytic cleavage of C-C bond in lignin models under visible light on mesoporous graphitic carbon nitride through π–π stacking interaction. ACS Catal.

[208] Shuai L, Amiri MT, Questell-Santiago YM, Héroguel F, Li Y, Kim H, Meilan R, Chapple C, Ralph J, Luterbacher JS (2016). Formaldehyde stabilization facilitates lignin monomer production during biomass depolymerization. Science.

[209] Lan W, Amiri MT, Hunston CM, Luterbacher JS (2018). Protection group effects during α, γ-diol lignin stabilization promote high-selectivity monomer production. Angew Chem Int Edit..

[210] Talebi Amiri M, Dick GR, Questell-Santiago YM, Luterbacher JS (2019). Fractionation of lignocellulosic biomass to produce uncondensed aldehyde-stabilized lignin. Nat Protoc.

[211] De Santi A, Galkin MV, Lahive CW, Deuss PJ, Barta K (2020). Lignin-first fractionation of softwood lignocellulose using a mild dimethyl carbonate and ethylene glycol organosolv process. Chemsuschem.

[212] Lai C, Yang B, He J, Huang C, Li X, Song X, Yong Q (2018). Enhanced enzymatic digestibility of mixed wood sawdust by lignin modification with naphthol derivatives during dilute acid pretreatment. Bioresour Technol..

[213] Pielhop T, Larrazábal GO, Studer MH, Brethauer S, Seidel C-M (2015). Rudolf von Rohr P: Lignin repolymerisation in spruce autohydrolysis pretreatment increases cellulase deactivation. Green Chem.

[214] Zhai R, Hu J, Saddler JN (2018). Minimizing cellulase inhibition of whole slurry biomass hydrolysis through the addition of carbocation scavengers during acid-catalyzed pretreatment. Bioresour Technol..

[215] Lai C, Jia Y, Zhou C, Yang C, Shen B, Zhang D, Yong Q (2020). Facilitating enzymatic digestibility of larch by in-situ lignin modification during combined acid and alkali pretreatment. Bioresour Technol..

[216] Questell-Santiago YM, Galkin MV, Barta K, Luterbacher JS (2020). Stabilization strategies in biomass depolymerization using chemical functionalization. Nat Rev Chem..

[217] Bertini F, Glatz M, Stöger B, Peruzzini M, Veiros LF, Kirchner K, Gonsalvi L (2019). Carbon dioxide reduction to methanol catalyzed by Mn(I) PNP pincer complexes under mild reaction conditions. ACS Catal.

[218] Jing Y, Guo Y, Xia Q, Liu X, Wang Y (2019). Catalytic production of value-added chemicals and liquid fuels from lignocellulosic biomass. Chem..

[219] Ferdosian F, Xu C (2017). Conversion of lignin into bio-based chemicals and materials.

[220] Wang Z, Qiu S, Hirth K, Cheng J, Wen J, Li N, Fang Y, Pan X, Zhu JY (2019). Preserving both lignin and cellulose chemical structures: flow-through acid hydrotropic fractionation at atmospheric pressure for complete wood valorization. ACS Sustain Chem Eng..

[221] Renders T, Van den Bosch S, Koelewijn SF, Schutyser W, Sels BF (2017). Lignin-first biomass fractionation: the advent of active stabilisation strategies. Energy Environ Sci..

[222] Sun Z, Fridrich B, de Santi A, Elangovan S, Barta K (2018). Bright side of lignin depolymerization: toward new platform chemicals. Chem Rev..

[223] Miyamoto T, Takada R, Tobimatsu Y, Takeda Y, Suzuki S, Yamamura M, Osakabe K, Osakabe Y, Sakamoto M, Umezawa T (2019). OsMYB108 loss-of-function enriches *p*-coumaroylated and tricin lignin units in rice cell walls. Plant J..

[224] Takeda Y, Suzuki S, Tobimatsu Y, Osakabe K, Osakabe Y, Ragamustari SK, Sakamoto M, Umezawa T (2019). Lignin characterization of rice coniferaldehyde 5-hydroxylase loss-of-function mutants generated with the CRISPR/Cas9 system. Plant J..

[225] Bryant ND, Pu Y, Tschaplinski TJ, Tuskan GA, Muchero W, Kalluri UC, Yoo CG, Ragauskas AJ (2020). Transgenic poplar designed for biofuels. Trends Plant Sci..

[226] Eudes A, Liang Y, Mitra P, Loqué D (2014). Lignin bioengineering.. Curr Opin Biotechnol.

[227] Brandon AG, Scheller HV (2020). Engineering of bioenergy crops: dominant genetic approaches to improve polysaccharide properties and composition in biomass. Front Plant Sci..

[228] Slewinski T (2012). Non-structural carbohydrate partitioning in grass stems: a target to increase yield stability, stress tolerance, and biofuel production. J Exp Bot..

[229] Li C, Chen C, Wu X, Tsang CW, Mou J, Yan J, Liu Y, Lin CSK (2019). Recent advancement in lignin biorefinery: with special focus on enzymatic degradation and valorization. Bioresour Technol..

[230] Sigoillot J-C, Berrin J-G, Bey M, Lesage-Meessen L, Levasseur A, Lomascolo A, Record E, Uzan-Boukhris E, Jouanin L, Lapierre C (2012). Chapter 8 - Fungal strategies for lignin degradation. Advances in Botanical Research.

[231] Dashtban M, Schraft H, Syed TA, Qin W (2010). Fungal biodegradation and enzymatic modification of lignin. Int J Biochem Mol Biol..

[232] Contreras F, Pramanik S, Rozhkova AM, Zorov IN, Korotkova O, Sinitsyn AP, Schwaneberg U, Davari MD (2020). Engineering robust cellulases for tailored lignocellulosic degradation cocktails. Int J Mol Sci..

[233] Dadwal A, Sharma S, Satyanarayana T (2020). Progress in ameliorating beneficial characteristics of microbial cellulases by genetic engineering approaches for cellulose saccharification. Front Microbiol..

[234] Davison SA, den Haan R, van Zyl WH (2020). Exploiting strain diversity and rational engineering strategies to enhance recombinant cellulase secretion by *Saccharomyces cerevisiae*. Appl Microbiol Biot..

[235] Sha C, Sadaqat B, Wang H, Guo X, Shao W (2020). Efficient xylan-to-sugar biotransformation using an engineered xylanase in hyperthermic environment. Int J Biol Macromol..

[236] Zhu L, Li P, Sun T, Kong M, Li X, Ali S, Liu W, Fan S, Qiao J, Li S (2020). Overexpression of* SFA1* in engineered *Saccharomyces cerevisiae* to increase xylose utilization and ethanol production from different lignocellulose hydrolysates. Bioresour Technol..

[237] Wei P, Lin M, Wang Z, Fu H, Yang H, Jiang W, Yang ST (2016). Metabolic engineering of Propionibacterium freudenreichii subsp shermanii for xylose fermentation. Bioresour Technol..

[238] Wang P, Zhang J, Feng J, Wang S, Guo L, Wang Y, Lee YY, Taylor S, McDonald T, Wang Y (2019). Enhancement of acid re-assimilation and biosolvent production in *Clostridium saccharoperbutylacetonicum* through metabolic engineering for efficient biofuel production from lignocellulosic biomass. Bioresour Technol..

[239] Fang D, Wen Z, Lu M, Li A, Ma Y, Tao Y, Jin M (2020). Metabolic and process engineering of for butyl acetate production in one step. J Agr Food Chem..

[240] Lee S-H, Yun EJ, Kim J, Lee SJ, Um Y, Kim KH (2016). Biomass, strain engineering, and fermentation processes for butanol production by solventogenic clostridia. Appl Microbiol Biot..

[241] Dai Z, Dong H, Zhu Y, Zhang Y, Li Y, Ma Y (2012). Introducing a single secondary alcohol dehydrogenase into butanol-tolerant *Clostridium acetobutylicum* Rh8 switches ABE fermentation to high level IBE fermentation. Biotechnol Biofuels..

[242] Kuehne SA, Minton NP (2012). ClosTron-mediated engineering of Clostridium. Bioengineered.

[243] Heap JT, Pennington OJ, Cartman ST, Carter GP, Minton NP (2007). The ClosTron: a universal gene knock-out system for the genus *Clostridium*. J Microbiol Meth..

[244] Shinto H, Tashiro Y, Kobayashi G, Sekiguchi T, Hanai T, Kuriya Y, Okamoto M, Sonomoto K (2008). Kinetic study of substrate dependency for higher butanol production in acetone–butanol–ethanol fermentation. Process Biochem..

[245] Sathesh-Prabu C, Kim D, Lee SK (2020). Metabolic engineering of *Escherichia coli* for 2,3-butanediol production from cellulosic biomass by using glucose-inducible gene expression system. Bioresour Technol..

[246] Zhao C, Chen S, Fang H (2018). Consolidated bioprocessing of lignocellulosic biomass to itaconic acid by metabolically engineering *Neurospora crassa*. Appl Microbiol Biot..

[247] Williamson JJ, Bahrin N, Hardiman EM, Bugg TDH (2020). Production of substituted styrene bioproducts from lignin and lignocellulose using engineered *Pseudomonas putida* KT2440. Biotechnol J..

[248] Mazzoli R (2020). Metabolic engineering strategies for consolidated production of lactic acid from lignocellulosic biomass. Biotechnol Appl Biochem.

[249] Chandel AK, Singh OV (2011). Weedy lignocellulosic feedstock and microbial metabolic engineering: advancing the generation of 'Biofuel'. Appl Microbiol Biot..

[250] Woo JE, Jang Y-S (2019). Metabolic engineering of microorganisms for the production of ethanol and butanol from oxides of carbon. Appl Microbiol Biotechnol..

[251] Cheng C, Bao T, Yang S-T (2019). Engineering *Clostridium* for improved solvent production: recent progress and perspective. Appl Microbiol Biotechnol.

[252] Agu CV, Ujor V, Ezeji TC (2019). Metabolic engineering of *Clostridium beijerinckii* to improve glycerol metabolism and furfural tolerance. Biotechnol Biofuels..

[253] Wang J, Zhang D, Chu F (2020). Wood-derived functional polymeric materials. Adv Mater..

[254] Kumar AK, Sharma S (2017). Recent updates on different methods of pretreatment of lignocellulosic feedstocks: a review. Bioresour Bioprocess..

[255] Sankaran R, Parra Cruz RA, Pakalapati H, Show PL, Ling TC, Chen WH, Tao Y (2020). Recent advances in the pretreatment of microalgal and lignocellulosic biomass: a comprehensive review. Bioresour Technol..

[256] Ali N, Zhang Q, Liu ZY, Li FL, Lu M, Fang XC (2020). Emerging technologies for the pretreatment of lignocellulosic materials for bio-based products. Appl Microbiol Biotechnol.

[257] Logeswaran J, Shamsuddin AH, Silitonga AS, Mahlia TMI (2020). Prospect of using rice straw for power generation: a review. Environ Sci Pollut Res..

[258] Chandra R, Takeuchi H, Hasegawa T (2012). Methane production from lignocellulosic agricultural crop wastes: a review in context to second generation of biofuel production. Renew Sust Energ Rev..

[259] Abu Bakar MS, Titiloye JO (2013). Catalytic pyrolysis of rice husk for bio-oil production. J Anal Appl Pyrol..

[260] Rahman IA (1992). Spherical gel particles from rice husk by chemical digestion. J Mater Chem..

[261] Hassan H, Lim JK, Hameed BH (2016). Recent progress on biomass co-pyrolysis conversion into high-quality bio-oil. Bioresour Technol..

[262] Wang X, Yang Z, Liu X, Huang G, Xiao W, Han L (2020). The composition characteristics of different crop straw types and their multivariate analysis and comparison. Waste Manag..

[263] Demirbaş A (2001). Relationships between lignin contents and heating values of biomass. Energy Convers Manage..

[264] Usmani Z, Sharma M, Gupta P, Karpichev Y, Gathergood N, Bhat R, Gupta VK (2020). Ionic liquid based pretreatment of lignocellulosic biomass for enhanced bioconversion. Bioresour Technol..

[265] Liang Y, Tang T, Umagiliyage AL, Siddaramu T, McCarroll M, Choudhary R (2012). Utilization of sorghum bagasse hydrolysates for producing microbial lipids. Appl Energy..

[266] Penín L, López M, Santos V, Alonso JL, Parajó JC (2020). Technologies for* Eucalyptus* wood processing in the scope of biorefineries: a comprehensive review. Bioresour Technol..

[267] González-García P (2018). Activated carbon from lignocellulosics precursors: a review of the synthesis methods, characterization techniques and applications. Renew Sustain Energy Rev..

[268] Tekin K, Karagöz S, Bektaş S (2012). Hydrothermal liquefaction of beech wood using a natural calcium borate mineral. J Supercrit Fluid..

[269] Park HJ, Dong J-I, Jeon J-K, Park Y-K, Yoo K-S, Kim S-S, Kim J, Kim S (2008). Effects of the operating parameters on the production of bio-oil in the fast pyrolysis of Japanese larch. Chem Eng J..

[270] Lama-Muñoz A, Del Mar CM, Espínola F, Moya M, Romero I, Castro E (2020). Characterization of the lignocellulosic and sugars composition of different olive leaves cultivars. Food Chem..

[271] Kang KE, Park D-H, Jeong G-T (2013). Effects of inorganic salts on pretreatment of* Miscanthus* straw. Bioresour Technol..

[272] Yang J, Xu H, Jiang J, Zhang N, Xie J, Zhao J, Bu Q, Wei M (2020). Itaconic acid production from undetoxified enzymatic hydrolysate of bamboo residues using *Aspergillus terreus*. Bioresour Technol..

[273] Alemán-Nava GS, Gatti IA, Parra-Saldivar R, Dallemand J-F, Rittmann BE, Iqbal HMN (2018). Biotechnological revalorization of tequila waste and by-product streams for cleaner production—a review from bio-refinery perspective. J Clean Prod..

[274] Weber B, Estrada-Maya A, Sandoval-Moctezuma AC, Martínez-Cienfuegos IG (2019). Anaerobic digestion of extracts from steam exploded *Agave tequilana* bagasse. J Environ Manage..

[275] Li H, Foston MB, Kumar R, Samuel R, Gao X, Hu F, Ragauskas AJ, Wyman CE (2012). Chemical composition and characterization of cellulose for Agave as a fast-growing, drought-tolerant biofuels feedstock. RSC Adv..

